# High-order photonic degeneracy enables topological reconfiguration in bound states in the continuum

**DOI:** 10.1038/s41467-026-75967-w

**Published:** 2026-07-22

**Authors:** Zhenchu Fu, Yang Chen, Fulong Shi, Rong Jin, Jiaoyang Guo, Yukang Zhang, Feilong Yu, Jin Chen, Lujun Huang, Chaobiao Zhou, Xiaoshuang Chen, Wei Lu, Andrea Alù, Guanhai Li, Cheng-Wei Qiu

**Affiliations:** 1 State Key Laboratory of Infrared Physics, Shanghai Institute of Technical Physics, Chinese Academy of Sciences, Shanghai, China; 2 School of Physical Science and Technology, ShanghaiTech University, Shanghai, China; 3 University of Chinese Academy of Sciences, Beijing, China; 4 Department of Precision Machinery and Precision Instrumentation, University of Science and Technology of China, Hefei, China; 5 Department of Electrical and Computer Engineering, National University of Singapore, Singapore, Singapore; 6 School of Physics and Electronic Science, East China Normal University, Shanghai, China; 7 College of Mechanical and Electronic Engineering, Guizhou Minzu University, Guiyang, China; 8 Hangzhou Institute for Advanced Study, University of Chinese Academy of Sciences, Hangzhou, China; 9 Shanghai Research Center for Quantum Sciences, Shanghai, China; 10 Photonics Initiative, Advanced Science Research Center, City University of New York, New York, NY USA

**Keywords:** Photonic crystals, Sub-wavelength optics

## Abstract

Extreme suppression of free-space radiation in open planar photonic systems remains a long-standing goal in nanophotonics. Conventional bound states in the continuum (BICs), constrained by symmetry-protected or accidental singularities, restrict experimental *Q*-**k** scaling exponents to *n* = 2-8 because ordinary bands lack sufficient degrees of freedom to host, redistribute or merge additional radiation-suppressing singularities. Here we demonstrate a degeneracy-driven topological-reconfiguration mechanism: an E_2_-type high-order degeneracy at Γ acts as a multi-charge reservoir, enabling controlled release, inversion and merging of topological charges under symmetry breaking. This unlocks a *Q* ∝ **k**^−10^ radiation-suppression regime in a single-layer all-dielectric metasurface. Experimentally, we realize ultrahigh free-space Q-factors with 3% geometric robustness and a maximum *Q* of 1.1 × 10^6^. A 100-pixel array exploiting these resonances demonstrates picometre-scale spectral discrimination by resolving ultranarrow H^13^C^14^N absorption lines. These results establish high-order-degeneracy-enabled BIC physics for robust ultrahigh-Q photonics and precision free-space spectroscopy.

## Abstract (web summary)

Bound states in the continuum provide high-quality resonances and ultrastrong light–matter coupling, yet their radiation suppression scaling is constrained by band topology. Here, the authors manipulate a high-order photonic degeneracy through engineered symmetry breaking in single-layer dielectric metasurfaces, providing additional control over topological charges.

## Introduction

The ability to suppress radiation loss in open planar photonic systems lies at the heart of modern nanophotonics^1–4^, enabling ultrahigh-Q resonances that amplify light–matter interactions^5–9^, sharpen spectroscopic selectivity^10–12^, and enhance nonlinear^13–16^ and quantum optical processes^17,18^. For nearly two decades, bound states in the continuum have served as a powerful route to achieving such extreme confinement under free-space excitation^19–22^, providing symmetry- or interference-enabled mechanisms for eliminating radiative leakage. Across metasurfaces, photonic crystals, and dielectric resonators, BICs have driven advances in sensing^23–26^, lasing^27–29^, nonlinear optics^30–33^, and topological photonics^34,35^. Nevertheless, despite these successes, a fundamental limit persists: the attainable Q-factor and its scaling with wavevector remain tightly constrained by the topological charge structures permitted in conventional photonic bands.

In traditional BIC platforms, radiative suppression is governed by the topological charge of the far-field polarization vortex associated with each singularity^36,37^. Symmetry-protected BICs—arising from group-theoretical radiation selection rules—typically host integer charges of ± 1 determined by the underlying point-group symmetry^31,38–40^. Accidental BICs, created by structural tuning, may introduce additional charges but remain confined to discrete configurations dictated by the available band topology. As a result, the slope of the experimental radiation-suppression scaling *Q* ∝ **k**^−n^ is limited to *n* = 2–6, reflecting the total charge that can be supplied or merged within a given band^28,41–43^. Recently, Kang et al. demonstrated that merging BICs with higher-order topological charges can extend this scaling to *Q* ∝ **k**⁻⁸, marking a significant advance in exploiting multi-charge interference for ultrahigh-Q metasurfaces^44^. As a result, the accessible topological charge space remains inherently constrained by the eigenmode structure of conventional photonic bands, which limits the extent to which radiation-suppressing singularities can be further hybridized.

To surpass this topological ceiling, it is necessary to access a qualitatively different class of photonic singularities—ones capable of generating and reconfiguring multiple charges within a single band. Unlike conventional band extrema, high-order degeneracies—being special topological singularities—inherently package more complex topological states^45–48^, offering a natural reservoir from which multiple radiation-suppressing singularities can emerge under symmetry breaking.

Here, we demonstrate that an E_2_ -type quadratic degeneracy at the Γ point enables precisely this functionality. By deliberately breaking the C_6_ symmetry of a single-layer all-dielectric metasurface, we trigger a controlled release, inversion, and merging of multiple integer and half-integer topological charges. This establishes a new topological pathway for BIC engineering beyond existing merging-BIC frameworks, in which additional charge families can be introduced and coherently combined. As a result, the scaling law is extended to *Q* ∝ **k**⁻¹⁰: starting from *Q* ∝ **k**⁻² for a single ±1 charge at Γ, each additional set of merged charges contributes a higher-order suppression term, leading to progressively enhanced radiation cancellation and structural robustness. Experimentally, this mechanism yields free-space Q-factors exceeding 1.1 × 10⁶ together with ~3% geometric tolerance. We further leverage these ultranarrow resonances in a 100-pixel metasurface array capable of picometer-scale spectral discrimination, validated using calibrated H¹³C¹⁴N absorption lines. These results reveal a new class of high-order-degeneracy–enabled BIC physics and provide a general pathway toward robust ultrahigh-Q photonics and precision free-space spectroscopy.

## Results

**Fig. 1:**
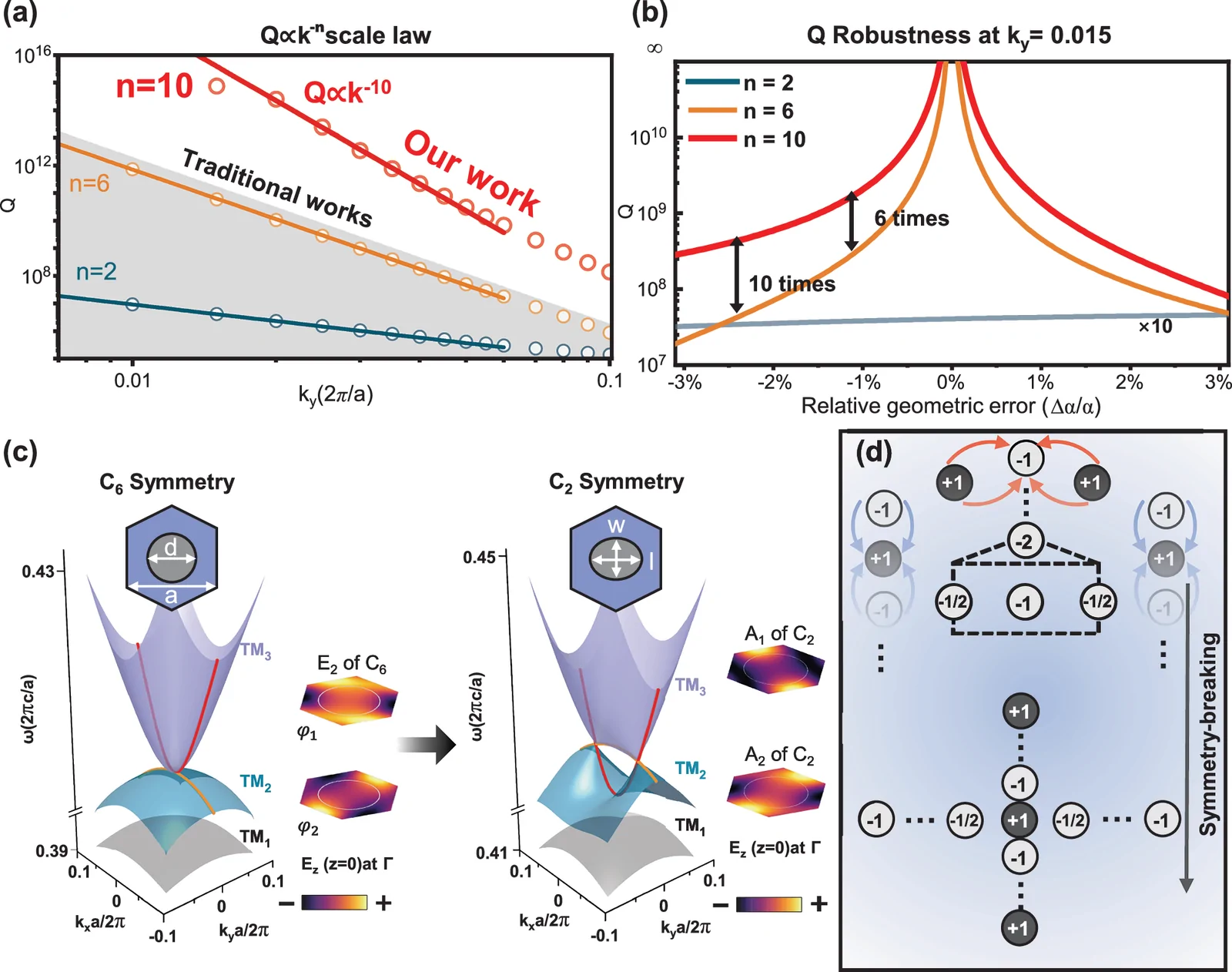
Scaling law and topological origin of ultrahigh-Q and robust BICs. **a** Comparison of *Q* ∝ **k**⁻ⁿ scaling laws arising from different topological mechanisms. The degeneracy-breaking strategy introduced here yields *n* = 10 (red line), far exceeding the limits of symmetry-protected (*n* = 2, green line) and hybrid accidental BICs (*n* = 6, orange line). **b** Free-space Q-factors under ±3% geometric perturbation. The degeneracy-engineered design retains nearly an order-of-magnitude higher Q than conventional merging-BIC platforms, demonstrating exceptional fabrication robustness. **c** Conceptual transition from a C_6_-symmetric photonic crystal unit cell to a perturbed C_2_ configuration. The lattice period is *a*, and the circular and elliptical holes are labeled by their diameter *d* and major/minor axis diameters *l/w*, respectively. The underlying Bloch band structure supports E₂ degeneracy at the Γ point, which is split under symmetry breaking. Insets show representative field profiles of the degenerate modes. **d** Far-field polarization vortex textures associated with the split modes. Symmetry-breaking releases and redistributes multiple integer and half-integer topological charges, while also inducing the reorganization of the outer accidental BICs, enabling the expanded and tunable radiation-suppression scaling observed in panel (**a**).

We consider a single-layer hexagonal photonic crystal slab whose unperturbed unit cell exhibits C₆ rotational symmetry (Fig. 1c). At the Γ point, the in-plane dipole modes form an E₂ representation, supporting a twofold (E₂) degeneracy at Γ point. Upon breaking the C₆ symmetry to C₂, this degeneracy is lifted, and the corresponding eigenmodes split into distinct radiative channels. This degeneracy lifting yields two key effects: First, it releases multiple topological charges into momentum space, which manifest as far-field polarization vortices with well-defined charge signs (Fig. 1d). Second, these charges—originating from both symmetry-protected and accidental mechanisms—can be spatially merged to induce constructive interference, enabling a new regime of radiation suppression that is both highly robust and tunable. During this process, the symmetry change drives the outer accidental BICs to redistribute and coalesce into new topological charges.

More specifically, the initial topological charge of – 2 at Γ can be interpreted as a composite of one integer charge (– 1) and two half-integer charges (– 1/2) associated with nearby Dirac points. Under symmetry breaking, this configuration evolves into a constellation of three integer (±1) charges and two half-integer (– 1/2) charges, distributed around Γ and along the k_y_-axis. This process occurs independently of external topological inputs and is decoupled from conventional accidental BIC tuning. Crucially, the ability to systematically release and reconfigure these multiple charges provides a previously inaccessible degree of topological control, enabling a record-high scaling law of *Q* ∝ **k**⁻¹⁰. This surpasses all prior BIC metasurface platforms, both in theoretical scaling exponent and experimentally measured Q-factor, and forms the topological foundation of our ultrahigh-Q yet fabrication-tolerant design.

**Fig. 2:**
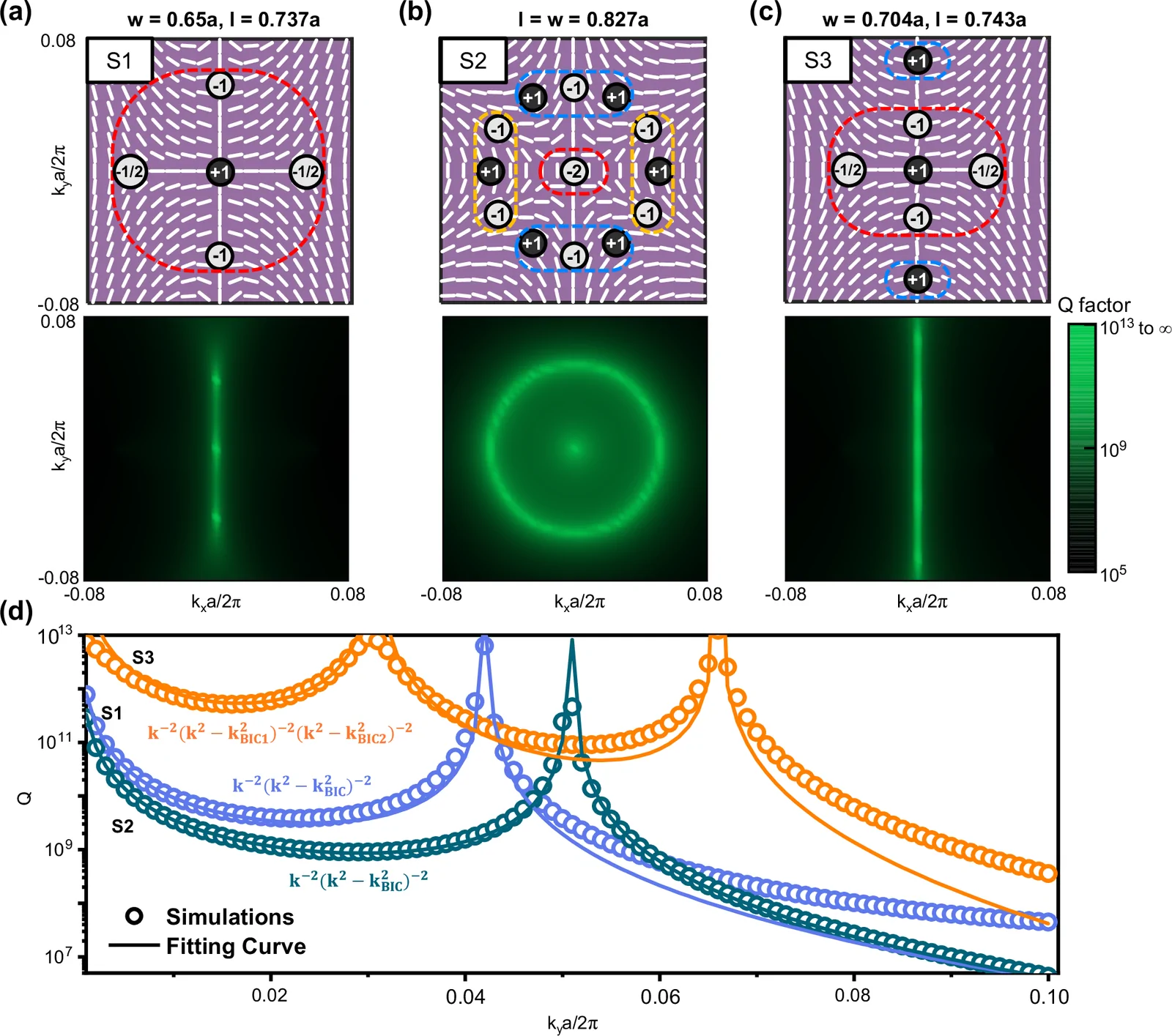
Controlled evolution of topological charges enables Q ∝ k⁻¹⁰ scaling. **a**–**c** Simulated far-field polarization textures and Q-factor maps of the TM₃ band under three representative unit-cell configurations. The dashed boxes in three different colors mark the evolution of different groups of topological charges under symmetry breaking. **a** Symmetry breaking lifts the E₂ degeneracy and releases multiple topological charges, including ± 1 and ± 1/2 vortices. **b** In the unperturbed C₆-symmetric structure, a central charge of – 2 appears at Γ, corresponding to a symmetry-protected BIC. **c** Combining symmetry breaking and accidental BIC engineering leads to three distinct topological charges aligned along the k_y_-axis. **d** Evolution of Q-factor scaling as multiple BICs approach Γ, transitioning from *Q* ∝ **k**⁻² to *Q* ∝ **k**⁻¹⁰. The three curves represent the Q-factor distributions in (**a**) purple curve, (**b**) green curve, and (**c**) orange curve, respectively.

To elucidate how high-order degeneracy enables steep Q-scaling, we track the evolution of topological charges in the TM₃ band as the unit-cell symmetry is gradually perturbed (Fig. 2a–c). The unperturbed structure, featuring C₆ symmetry with circular holes, exhibits an E₂-degenerate dipole mode at Γ, giving rise to a symmetry-protected BIC with topological charge – 2 and a scaling law of *Q* ∝ **k**⁻². This scaling is weaker than the conventional *Q* ∝ **k**⁻⁴, due to the additional vortex order contributed by the degeneracy point (see Supplementary Note 1 with Supplementary Fig. 1, 2 and Supplementary Table S1).

Upon breaking C₆ to C₂ symmetry by deforming the holes into ellipses, the E₂ degeneracy is lifted and the original – 2 charge splits into multiple vortex singularities (Fig. 2a). These include two – 1/2 charges at the split degeneracy points and a pair of – 1 charges migrating along the k_y_-axis. Simultaneously, the central Γ-point charge reverses sign to + 1, conserving the total topological charge. At this stage, the accidental BICs are located far away from the center, allowing the evolution of the topological charge at the central degeneracy point to be displayed clearly in isolation. Such rich charge dynamics—enabled by the multi-mode nature of high-order DPs—are inaccessible in conventional single-mode BIC systems so far (see Supplementary Note 2 and Supplementary Fig. 3).

Without breaking the structural symmetry, increasing the hole radius continuously tunes the interference condition of the radiative channels, driving the accidental BICs from an outer ring toward the vicinity of the Γ point. Figure 2b shows the corresponding topological-charge distribution, where these accidental BICs are symmetrically distributed along six high-symmetry lines, forming a ring-like configuration. These charges form a controllable reservoir of off-Γ charges.

By subsequently introducing symmetry breaking, the degeneracy at Γ is lifted, and additional topological charges are released from the high-order degenerate point, while the pre-existing outer-ring charges are simultaneously redistributed in momentum space. As a result, the final topological configuration can be deterministically selected by controlling the sequence of structural tuning and symmetry reduction. For clarity, all groups of charges involved in this process—including the outer-ring accidental charges and the degeneracy-released charges—are highlighted in Fig. 2a–c using dashed boxes with different colors. In particular, the + 1 charges appearing at the top and bottom in Fig. 2c originate from the merging of multiple contributions, the corresponding charges are marked by the same blue dashed boxes in Fig. 2b. Further theoretical details, additional calculations, and high-resolution polarization-vortex maps illustrating the full topological evolution are provided in Supplementary Note 3.

As the geometry is further tuned (*w*  =  0.704a, *l*  =  0.743a), the released charges spatially converge near Γ, forming three distinct BICs aligned along the k_y_-axis (Fig. 2c). This aggregation sets the stage for constructive interference among multiple radiation-suppressed states. The resulting Q-factor scaling evolution is shown in Fig. 2d. Initially, the system follows *Q* ∝ **k**⁻². As the BICs approach Γ, the scaling transitions to a composite form incorporating their individual contributions. When all BICs coalesce near Γ (e.g., *w* = 0.721*a*, *l* = 0.752*a*, provided in Supplementary Fig. S4), the full topological merging leads to a scaling law of *Q* ∝ **k**⁻¹⁰, as derived analytically in Supplementary Note 4. Notably, in previously reported higher-order merging scenarios (e.g., *Q* ∝ **k**⁻⁸), the scaling enhancement is achieved through the merging of charges confined within a fixed band topology. Here, the involvement of a high-order degenerate point acts as an additional charge reservoir, allowing independent charge generation and reconfiguration, and thereby enabling a higher-order scaling of *Q* ∝ **k**⁻¹⁰. Beyond scaling, the spatial arrangement of BICs also enables control over the angular bandwidth of the high-Q region (see Supplementary Note 5 and Supplementary Fig. 5), offering a new degree of design freedom.

**Fig. 3:**
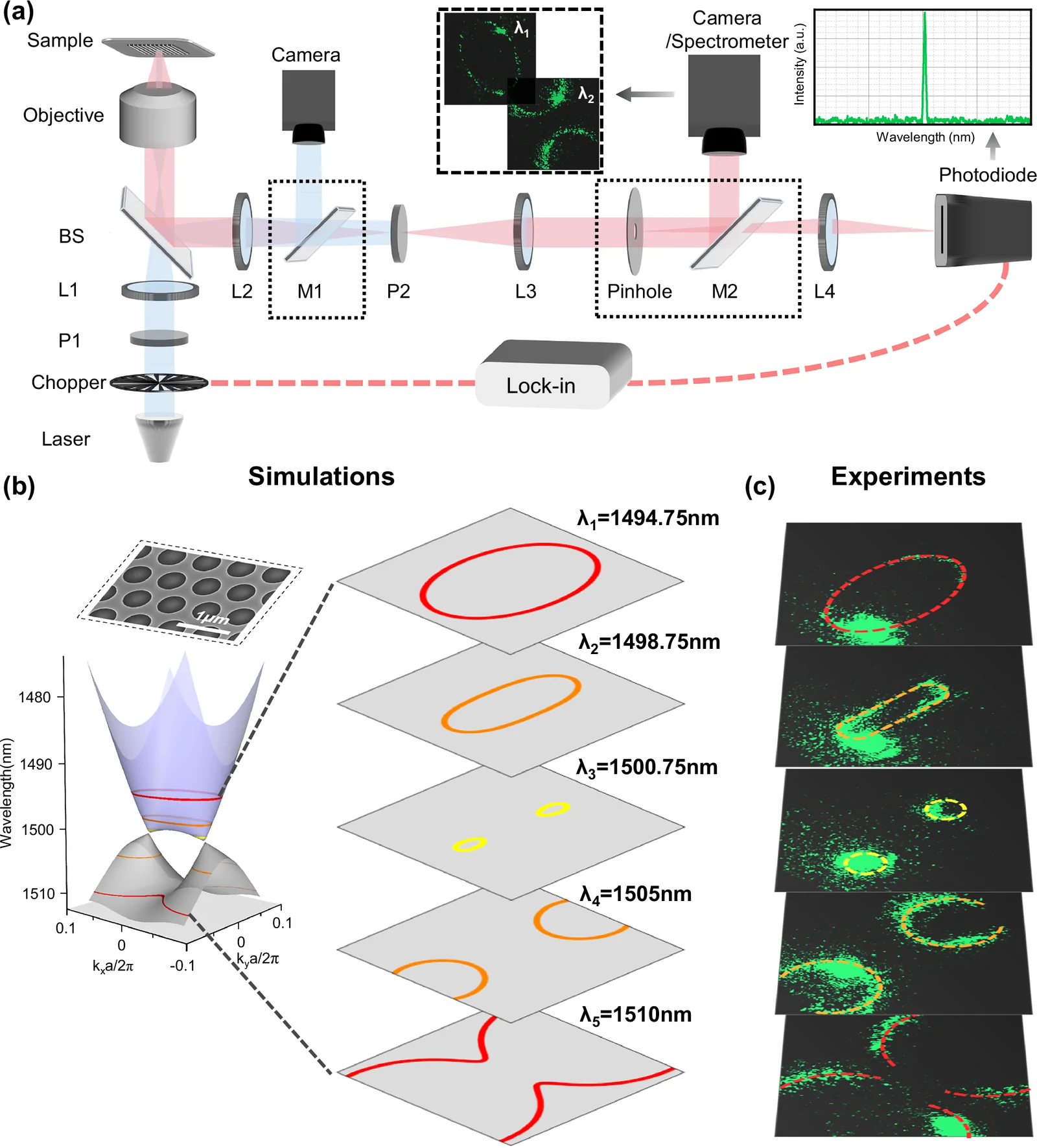
Experimental observation of momentum-space topological evolution near high-order degeneracy. **a** Schematic of the resonance-enhanced scattering setup with cross-polarized detection, enabling acquisition of both high-Q resonance spectra and angular-resolved isofrequency contours. Insets show representative results under spectral (left) and angular (right) detection modes. **b** Simulated photonic band structure and five isofrequency contours from the TM₃ band near the degeneracy point. **c** Measured isofrequency contours at corresponding wavelengths, revealing excellent agreement with simulations and tracking the continuous evolution and splitting of the high-order degeneracy.

To experimentally probe the momentum-space signatures of high-order degeneracy lifting, we employ a custom-built cross-polarization measurement system based on resonance-enhanced scattering (Fig. 3a; see “Methods”). This setup enables both spectral and angular resolution, allowing direct mapping of isofrequency contours associated with high-Q resonances. Figure 3b shows the simulated band structure and five representative isofrequency contours of the TM₃ band near the degenerate point. The corresponding experimental contours are presented in Fig. 3c, exhibiting excellent agreement in both shape and evolution. At shorter wavelengths (e.g., 1494.75 nm), the contours appear as large ellipses centered at Γ. As the wavelength increases, the contours contract and split into two localized regions around the lifted degeneracy points (e.g., at 1500.75 nm). At longer wavelengths, the contours expand again and form a ring-like structure. This spectral progression visualizes the dynamic redistribution of far-field signatures. The clear match between simulation and measurement confirms the geometric tunability and robustness of the degeneracy-breaking design. Notably, these features remain observable despite fabrication imperfections, underscoring the topological protection embedded in our multi-BIC merging mechanism.

**Fig. 4:**
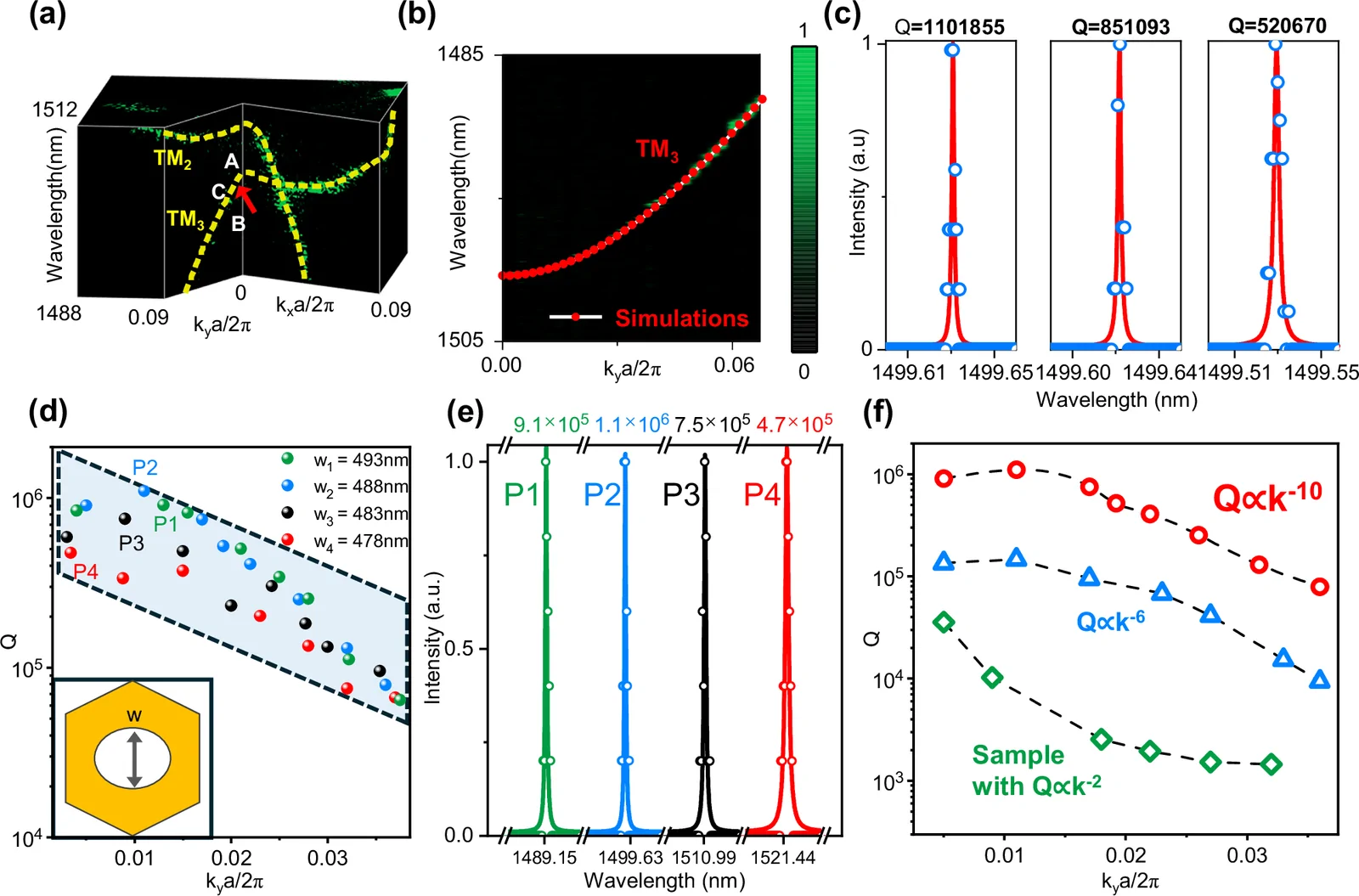
Experimental validation of ultrahigh-Q resonances and fabrication robustness. **a** Three-dimensional reconstruction of isofrequency contours from angular-resolved scattering, visualizing band crossings and field suppression due to topological charge interference. **b** Measured angle-resolved spectrum along the k_y_-axis (gray map), overlaid with simulated eigenfrequencies (red dots), showing strong agreement. **c** Extracted scattering spectra at three momentum points (A–C) near Γ, all exhibiting Q > 10⁵. Point A achieves Q = 1.1 × 10⁶. **d** Q-factor distribution across samples with varied short-axis widths (3%). All samples maintain Q > 10^5^, highlighting fabrication tolerance. Inset: schematic of unit cell geometry. **e** Measured spectra at the highest-Q points (p1–p4) from the sample set in (**d**) illustrating robust performance across fabrication variations. **f** Comparison of Q-factor distributions for three design strategies. Our degeneracy-breaking design (red) delivers the highest Q and broadest angular bandwidth.

To experimentally validate the ultrahigh-Q resonances and robustness of our degeneracy-breaking BIC design, we performed angular- and position-resolved scattering measurements. Figure 4a presents a 3D reconstruction of the isofrequency contours near the degeneracy point, obtained by stacking CCD images at different angles. The mapped contours reveal band crossings and suppressed radiation leakage—hallmarks of topological charge interference. Figure 4b shows the measured angle-resolved spectrum along the k_y_-axis, overlaid with simulated eigenfrequencies (red dots). The close match confirms the predictive accuracy of our theoretical model. At three representative momentum points near Γ (A–C), Lorentzian fitting yields Q-factors all above 10⁵, with point A reaching 1.1 × 10⁶ (Fig. 4c). These results confirm that high-Q states are not confined to isolated symmetry points but extend across a continuous angular region—direct evidence of the multi-BIC merging mechanism in action.

We fabricated a series of devices with 3% variation in elliptical short-axis width to assess the fabrication tolerance (Fig. 4d). All samples maintain Q > 10⁵, and the highest Q again exceeds 10⁶, underscoring the resilience of the design to geometric disorder (Details are provided in Supplementary Notes 6 and 7, Supplementary Figs. 6–9). Representative spectra from each sample (Fig. 4e) further demonstrate reproducibility and robustness. Finally, we benchmarked our design against two established BIC strategies: (i) a single symmetry-protected BIC, and (ii) a conventional merging-BIC system. As shown in Fig. 4f, our approach achieves both the highest peak Q and the widest angular high-Q window. For the merging-based designs (Q ∝ **k**⁻⁶ and Q ∝ **k**⁻¹⁰), the maximum Q does not necessarily occur at the smallest **k**; instead, it reflects the combined effect of finite intrinsic losses and slight deviations from the ideal merging condition, which can shift the location of the maximum Q factor in practical experiments. These results not only validate the Q ∝ **k**⁻¹⁰ scaling but also demonstrate practical advantages for scalable fabrication.

**Fig. 5:**
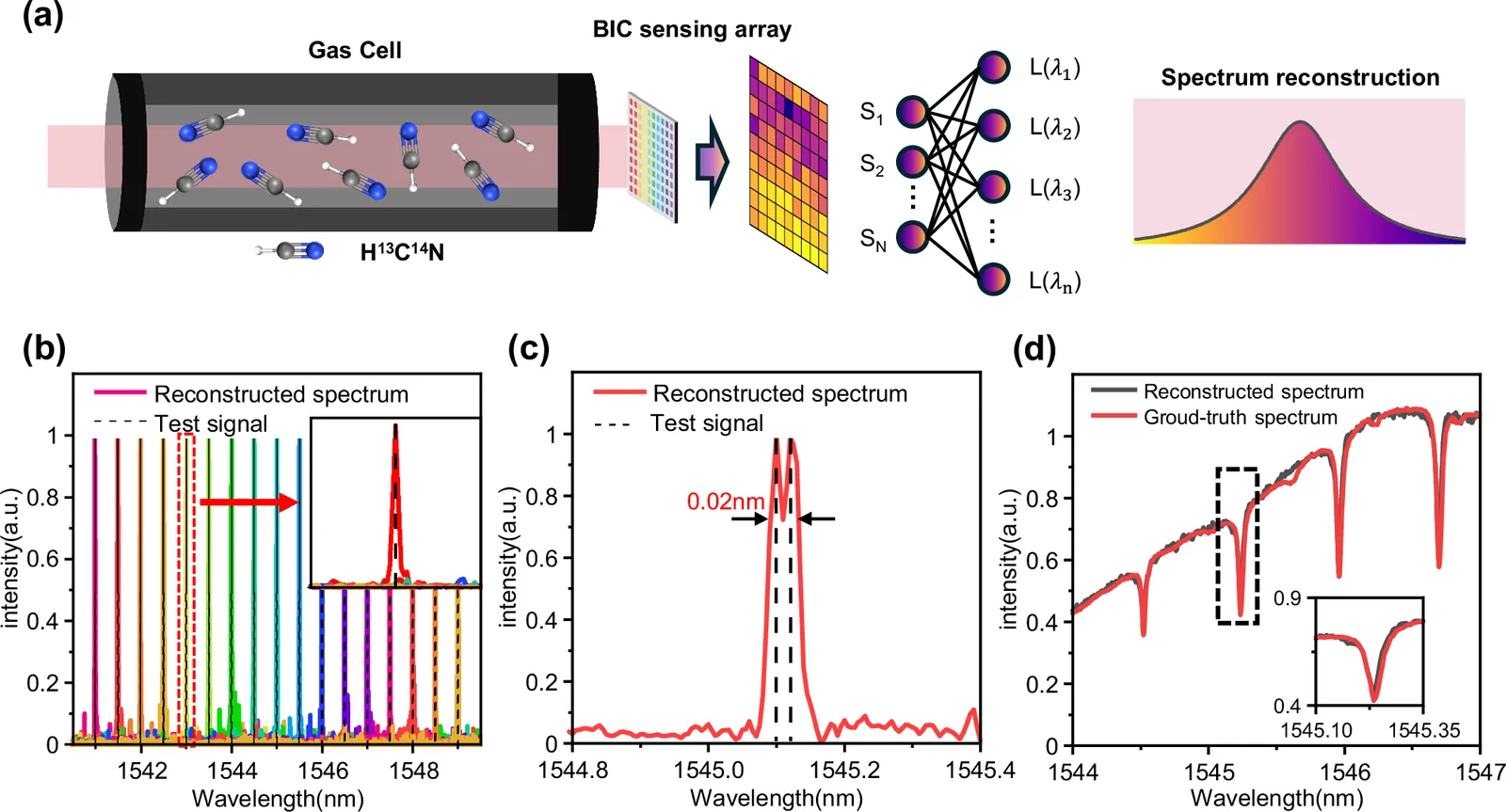
Free-space gas-sensing demonstration enabled by an ultrahigh-Q metasurface array. **a** Schematic of the free-space sensing platform integrating a 100-pixel ultrahigh-Q metasurface array with a neural-network–based spectral reconstruction module. Measurements were performed using H^13^C^14^N at room temperature and 50 Torr with a 16.5 cm optical path. **b** Reconstructed spectra from experimental monochromatic inputs (linewidth 200 kHz) show near-perfect alignment with target wavelengths, with FWHM ~ 0.01 nm. **c** Resolution test with two spectral lines spaced 0.02 nm apart, output from the laser, clearly resolved by the developed system. **d** Measured H¹³C¹⁴N absorption spectrum (red) compared with the reconstructed spectrum (gray), demonstrating high-fidelity retrieval of ultranarrow reference lines and validating picometer-scale discrimination under free-space excitation.

The ultrahigh-Q resonances provided by the degeneracy-engineered metasurface offer a natural foundation for precision free-space spectroscopic sensing. We construct a chip-scale spatial–spectral array comprising 100 individually engineered metasurface pixels (Fig. 5a), each supporting an ultranarrow resonance arising from the controlled merging of multiple BIC charges. This configuration enables dense wavelength encoding directly in free space, without waveguides or coupling structures. The global intensity pattern formed across the array is decoded using a deep neural network (see Methods and Supplementary Note 8 with Supplementary Figs. 10–11), which reconstructs incident spectra with high fidelity.

Prior to gas measurements, we calibrated the system using a tunable laser with a 200 kHz linewidth. The reconstructed spectra (Fig. 5b) exhibit accurate wavelength retrieval with a full-width-at-half-maximum of ~ 0.01 nm—comparable to bulk cavity systems. To assess the intrinsic resolution, we injected two spectral lines separated by 0.02 nm; the system cleanly resolves both peaks (Fig. 5c), exceeding the resolving power of most integrated or grating-based spectrometers operating in this band (see Supplementary Note 9 and Supplementary Table S2).

To benchmark sensing accuracy, we measured the absorption spectrum of hydrogen cyanide (H^13^C^14^N), a widely used wavelength-reference gas with well-tabulated transitions in the telecommunication band. This choice enables a calibrated assessment of picometer-scale discrimination. Under a 16.5 cm path length at 50 Torr, the reconstructed absorption profile (Fig. 5d) matches the reference spectrum with high fidelity, accurately recovering both line centers and relative amplitudes. The excellent agreement—together with the demonstrated 0.02-nm resolution and ~ 0.01-nm linewidth recovery—confirms that the metasurface array achieves reliable picometer-scale sensing under free-space excitation. These results highlight that a simple single-layer architecture can deliver performance—spectral precision, robustness, accuracy, and compactness—that meets or surpasses state-of-the-art free-space or integrated spectrometers.

## Discussion

In summary, we have shown that a high-order photonic degeneracy provides a powerful and previously inaccessible route for engineering the topology of bound states in the continuum. By lifting an E_2_ -type quadratic degeneracy through controlled symmetry breaking, we unlock a mechanism capable of releasing, reconfiguring, and merging multiple topological charges within a single photonic band—thereby establishing a new route for topological control of BICs beyond conventional merging schemes. In this framework, the involvement of degeneracy-enabled charge reservoirs expands the accessible topological phase space, allowing additional groups of accidental BICs to be independently generated and coherently combined. This leads to an extended scaling law of Q ∝ **k**⁻¹⁰ in a single-layer all-dielectric metasurface, together with experimentally demonstrated free-space Q-factors exceeding 1.1 × 10⁶ and robust performance under ~3% geometric variations. These ultranarrow, fabrication-robust resonances naturally translate into precision free-space spectroscopy, as demonstrated through picometer-scale discrimination of calibrated H^13^C^14^N absorption lines using a 100-pixel metasurface array.

Beyond this specific implementation, our results identify high-order photonic degeneracies as a general and scalable means of accessing new topological degrees of freedom, expanding the landscape of BIC physics. This framework opens opportunities for ultrahigh-Q metasurfaces, long-coherence photonic states, enhanced nonlinear and quantum interactions, and new regimes of radiation control that transcend the constraints of conventional band topology.

## Methods

### Simulations

All numerical simulations in this work are carried out with the commercial finite-element package COMSOL Multiphysics. Bloch-periodic boundary conditions are applied in the x-y plane, whereas perfectly matched layers (PMLs) are used along the z-direction. In Fig. 1 the reference structures with scaling exponents *n* = 2 and *n* = 6 are taken from refs. ^31,42^, respectively. For Fig. 1b, we scan the same geometric parameters as in the original reports, namely the block side length, the lattice period, and the hole diameter.

### Sample fabrication

All metasurfaces were fabricated on a silicon-on-insulator wafer (thickness *h* = 430 nm). A 330 nm-thick ZEP520 photoresist layer was spin-coated and patterned via electron-beam lithography. The patterns were transferred into the silicon layer by inductively coupled plasma etching using a Die-RIE-Oxford-80 system. Post-etching, residual resist was removed in N-methyl-2-pyrrolidone. To ensure up-down mirror symmetry, the buried oxide layer was subsequently removed with hydrofluoric acid. The photonic crystal lattice constant was set to *a* = 677 nm. Elliptical air holes were defined with a fixed aspect ratio *l/w* = 1.042, while the short axis *w* was varied across samples to tune the degeneracy-breaking condition.

### Optical characterization

Measurements were performed using a tunable telecom laser source (Santec TSL-550, 1480–1630 nm), which provides a wavelength tuning precision of 0.0001 nm and a linewidth of approximately 200 kHz (≈0.00016 nm). The incident beam was linearly polarized (Glan-Taylor polarizer) and focused onto the back focal plane of a high-NA objective lens to control the incident in-plane momentum. The reflected signal was collected by a photodiode (PDA10DT-EC), while angular selectivity was achieved by inserting a movable pinhole at the Fourier plane. Isofrequency contours were recorded using a CCD camera (Hamamatsu C1274-03). A crossed-polarization detection scheme was employed to suppress background reflections. To compensate for the inherently low signal intensity, we used a lock-in amplifier (SR830) synchronized with a mechanical chopper for enhanced SNR. For measurements on the substrate-removed samples, the much weaker scattering signal required step-scan point-by-point acquisition and a longer (300 ms) lock-in integration time.

### Neural network setup

A fully connected DNN was trained to reconstruct spectra from 100 metasurface pixel responses. Each input vector was normalized and expanded with pairwise multiplicative terms to capture higher-order correlations, resulting in a 1717-dimensional input space. The network consists of three hidden layers with 2800, 3500, and 2800 neurons, respectively, using ReLU activation. The output layer is linear to preserve spectral amplitudes. Training was conducted using the Adam optimizer with L2 regularization, minimizing the mean-squared error (MSE) over 1.5 × 10⁵ iterations. The metasurface array used for data acquisition included slight variations in lattice periodicity and elliptical short-axis lengths, while maintaining a constant device-layer thickness and elliptical aspect ratio *l/w* = 1.044. The central parameters are set to lattice periodicity *a* = 677 nm, thickness *h* = 448 nm, and elliptical short-axis lengths *w* = 478.2 nm.

### Gas measurements

The standard H^13^C^14^N reference gas cell was sourced from Wavelength References, LLC. Because H^13^C^14^N is highly toxic, the cell was vendor-sealed and custom specified. We selected 50 Torr (~6.7 kPa) and a 16.5 cm optical path length to match the system precision and enable limit testing. Calculations based on the HITRAN database indicate an absorption linewidth of approximately 0.03 nm under these conditions. The absorption spectrum was measured in the original system optical path and compared with the reconstructed spectrum to verify the accuracy of spectral reconstruction. Line centers were cross-validated against the HITRAN database to avoid systematic bias.

## Supplementary information


Supplementary Information
Transparent Peer Review file


## Data Availability

The data supporting the findings of this study are provided in the main text and supplementary information file and at the Zenodo database (https://doi.org/10.5281/zenodo.20794040).
