# Supplementary material for: High-order photonic degeneracy enables topological reconfiguration in bound states in the continuum
Source: Nat Commun. 2026 Jul 22;17:9203. doi: 10.1038/s41467-026-75967-w (PMC13522528; doi:10.1038/s41467-026-75967-w)
Supplement: Supplementary file 1 [file 41467_2026_75967_MOESM1_ESM.pdf]

Supplementary Material for

High-Order Photonic Degeneracy Enables Topological Reconfiguration in Bound States in the Continuum

Zhenchu Fu[1,2,3+], Yang Chen[4+], Fulong Shi[5+], Rong Jin[1,3], Jiaoyang Guo[1,3], Yukang Zhang[1,3], Feilong Yu[1,3], Jin Chen[1,3], Lujun Huang[6], Chaobiao Zhou[7], Xiaoshuang Chen[1,3,8,9], Wei Lu[1,3,8,9], Andrea Alu[10*], Guanhai Li[1,3,8,9*], Cheng-Wei Qiu[5*]

[1]State Key Laboratory of Infrared Physics, Shanghai Institute of Technical Physics, Chinese Academy of Sciences, 500 Yu Tian Road, Shanghai, 200083, China

[2]School of Physical Science and Technology, ShanghaiTech University, 393 Mid Huaxia Road, Shanghai, 201210, China

[3]University of Chinese Academy of Sciences, No. 19 Yuquan Road, Beijing, 100049, China

[4]Department of Precision Machinery and Precision Instrumentation, University of Science and Technology of China, 230027 Hefei, China

[5]Department of Electrical and Computer Engineering, National University of Singapore, 4 Engineering Drive 3, Singapore 117583, Singapore

[6]School of Physics and Electronic Science, East China Normal University, Shanghai, 200241, China

[7]College of Mechanical and Electronic Engineering, Guizhou Minzu University, Guiyang, 550025, China

[8]Hangzhou Institute for Advanced Study, University of Chinese Academy of Sciences, No.1 SubLane Xiangshan, Hangzhou, 310024, China

[9]Shanghai Research Center for Quantum Sciences, 99 Xiupu Road, Shanghai, 201315, China

[10]Photonics Initiative, Advanced Science Research Center, City University of New York, New York, NY, 10031, USA

+ *Zhenchu Fu, Yang Chen and Fulong Shi contributed equally:*

* *chengwei.qiu@nus.edu.sg; ghli0120@mail.sitp.ac.cn, aalu@gc.cuny.edu*

Supplementary Note 1: Topological Nature of High-order Degenerate BICs

The topological charge of polarization singularities in momentum space is defined by the following equation:

$$q = \frac{1}{2\pi}\oint_C d\mathbf{k}\cdot\Delta_{\mathbf{k}}\varphi(\mathbf{k}) \qquad (S.1)$$

Here C is a closed path in momentum space, and $\varphi(\mathbf{k}) = \arg\,[c_x(k) + ic_y(k)]$ is the polarization angle phase of the far-field polarization vector. The topological charge quantifies the number of cycles of phase change around the singularity. The polarization vector $\mathbf{c}(\mathbf{k})$ is function of reciprocal space vector $\mathbf{k}$. By constraining the phase of the eigenfunction, $\mathbf{c}(\mathbf{k})$ becomes purely real and continuous with respect to $\mathbf{k}$, with its symmetry dictated by the point group of the structure. For a structure with in-plane point group symmetry $\varepsilon(\mathbf{r}) = \varepsilon(R\mathbf{r})$, the polarization vector satisfies $\mathbf{c}(\mathbf{k}) = \alpha R\mathbf{c}(R^{-1}\mathbf{k})$. Due to the continuity of $\mathbf{c}(\mathbf{k})$, α must be either +1 or -1 for a given band under symmetric operations. Thus, the irreducible representation of the mode at the Γ point determines its topological charge and the nearby polarization field distribution.

Nevertheless, the foregoing approach is only valid for non-degenerate photonic bands. For degenerate bands, the degeneracy point represents a singularity where the mode is discontinuous with the surrounding states. Therefore, different considerations are needed for the polarization vortex configuration in degenerate bands.

By removing the central degeneracy point, $\mathbf{u}(\mathbf{k})$ and $\mathbf{c}(\mathbf{k})$ remain continuous in other regions of the band, still satisfying $\mathbf{c}(\mathbf{k}) = \alpha R\mathbf{c}(R^{-1}\mathbf{k})$, with α remaining a constant. Therefore, $\mathbf{c}(\mathbf{k})$ can be characterized by a one-dimensional irreducible representation of the structural point group. The challenge lies in relating this to the two-dimensional irreducible representation of the degenerate mode. Our conclusion is that the eigenvalues of $\mathbf{c}(\mathbf{k})$ and the central degenerate mode are opposite under the C_2 symmetric operation. This is derived from the point group character table, where C_2 is the only symmetry of the degenerate mode, except in the C_3 symmetry system.

The quadratic degeneracy structure around the Γ point can be seen as the superposition of two identical Dirac degeneracies, each contributing half-integer charges that lead to distinct polarization characteristics inside and outside the degeneracy point. To understand this, we introduce a small perturbation H', breaking the symmetry to C_2, as shown in Fig. S1a. Although this perturbation barely affects the band structure, it causes the degeneracy point to split into two Dirac points. The degenerate mode also splits into two sub-modes, which can be characterized using

group theory's compatibility principle. This yields the relationship between the symmetry of $\mathbf{c}(\mathbf{k})$ for the degenerate band and the degenerate mode at the Γ point. Fig. S1b shows that the characteristics of region II remain unchanged after introducing the perturbation, resulting in the following relations:

$$\mathbf{c}(\mathbf{k}_\mathrm{A}) = \alpha_1 \mathbf{C_2} \mathbf{c}(\mathbf{k}_\mathrm{D}) \qquad (S.2)$$

In the continuous internal region I, similar to a singlet state, we have:

$$\mathbf{c}(\mathbf{k}_\mathrm{B}) = \alpha_\Gamma \mathbf{C_2} \mathbf{c}(\mathbf{k}_\mathrm{C}) \qquad (S.3)$$

The symmetry coefficient in this region is determined by the irreducible representation of the mode at the Γ point.

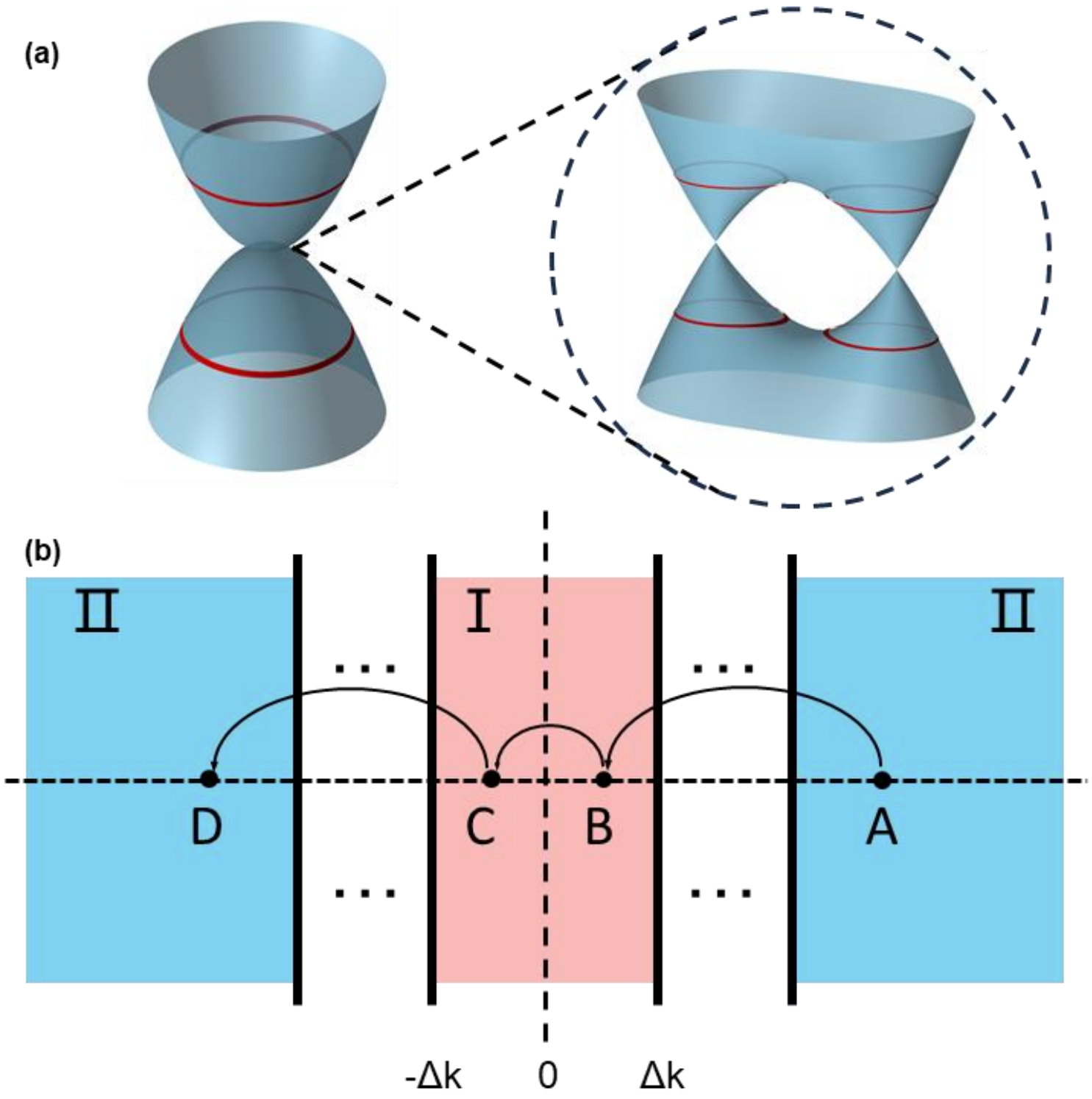

Supplementary Fig. 1. Structure of degeneracy band eigenstates. a, The quadratic degeneracy can be regarded as the superposition of two Dirac degeneracies. **b**, After introducing a perturbation, momentum space is divided into two regions: region I, near the degeneracy point, and region II, far from it. The blue region corresponds to the unperturbed band state, while the red region represents the internal band state after degeneracy point splitting.

It has been previously established that Dirac points contribute half-integer charges. In our analysis, as long as the path does not encircle the Dirac point, the change in $\mathbf{c}(\mathbf{k})$ remains continuous without causing polarization reversal. Thus, the phase change can be examined along the path A-B-C-D in Fig. S1b. Defining the phase change along the path $\mathbf{S}$ as:

$$f(S) = \int_S d\mathbf{k} \cdot \Delta_{\boldsymbol{k}} \varphi(\mathbf{k}) \quad (S.4)$$

From Eq. S.3, we obtain:

$$f(B \rightarrow C) = 2n\pi \text{ or } (2n+1)\pi \ n \in Z \quad (S.5)$$

For the phase change from A to D:

$$f(A \rightarrow D) = f(A \rightarrow B) + f(B \rightarrow C) + f(C \rightarrow D) \quad (S.6)$$

Due to the C2 symmetry, $f(A \rightarrow B)$+ $f(C \rightarrow D)$ forms a closed integral around a Dirac point , leading to:

$$f(A \rightarrow D) = f(B \rightarrow C) + (2n+1)\pi \ n \in Z \quad (S.7)$$

Thus, the phase change from A to D is (2n+1)π greater than from B to C, indicating that $\alpha_1 = -\alpha_\Gamma$. For structures with mirror symmetry, the polarization transformation across the degeneracy point confirms that the mirror symmetry coefficient is also reversed. These relationships establish the symmetry properties of the polarization vortex associated with the degeneracy band.

Taking the C_{6v} system as an example, the two-dimensional irreducible representation E_2 under C_2 operation is 2, indicating that the degenerate field corresponds to even modes. Under C_2, E_2 splits into $A_1 \oplus A_2$ mode, both even under C_2. According to our theory, the two $\mathbf{c(k)}$ modes on this degenerate band are odd under C_2, with different mirror symmetries. Thus, $\mathbf{c(k)}$ for the upper and lower bands belongs to the B_1 and B_2 representations of the C_{6v} point group, allowing for charges of -2+6n. As mentioned above, after removing the degeneracy point, $\mathbf{c(k)}$ on the two bands can be characterized by a one-dimensional irreducible representation under the point group of structure. The C_3 point group has only one one-dimensional irreducible representation, so the degeneracy band is consistent with the singlet band from the topological configuration of $\mathbf{c(k)}$. Based on the above research, we study the allowed topological charges under different symmetric structures and summarize them in Table 1. Then we calculate the polarization configurations of degeneracy bands with different structures and show them in Fig. S2. All calculations are based on the first degeneracy band of silicon structure TM mode. The period of all structures is a, the thickness of slabs is $0.7a$, and the air-hole diameter is $0.6a$. The side length of the triangle hole is $0.66a$. The results are consistent with our theory. Similar analyses apply to other degenerate modes, with the possible charges summarized in Table S1. Figure S2 shows the calculated polarization configurations of degeneracy bands for different structures, consistent with

theoretical predictions.

Table S1. Allowed charges for all bands, indicating whether each mode supports symmetry-protected BICs.

Symmetry	Representation	Allowed charges	BIC(Y/N)
C_{2v}	A_1, A_2	±1+2n	Y
	B_1, B_2	2n	N
C_{3v}	A_1, A_2	1+3n	Y
	E	1+3n	N
C_{4v}	A_1, A_2	1+4n	Y
	B_1, B_2	-1+4n	Y
	E	±1+4n	N
C_{6v}	A_1, A_2	1+6n	Y
	B_1, B_2	-2+6n	Y
	E_1	1+6n	N
	E_2	-2+6n	Y

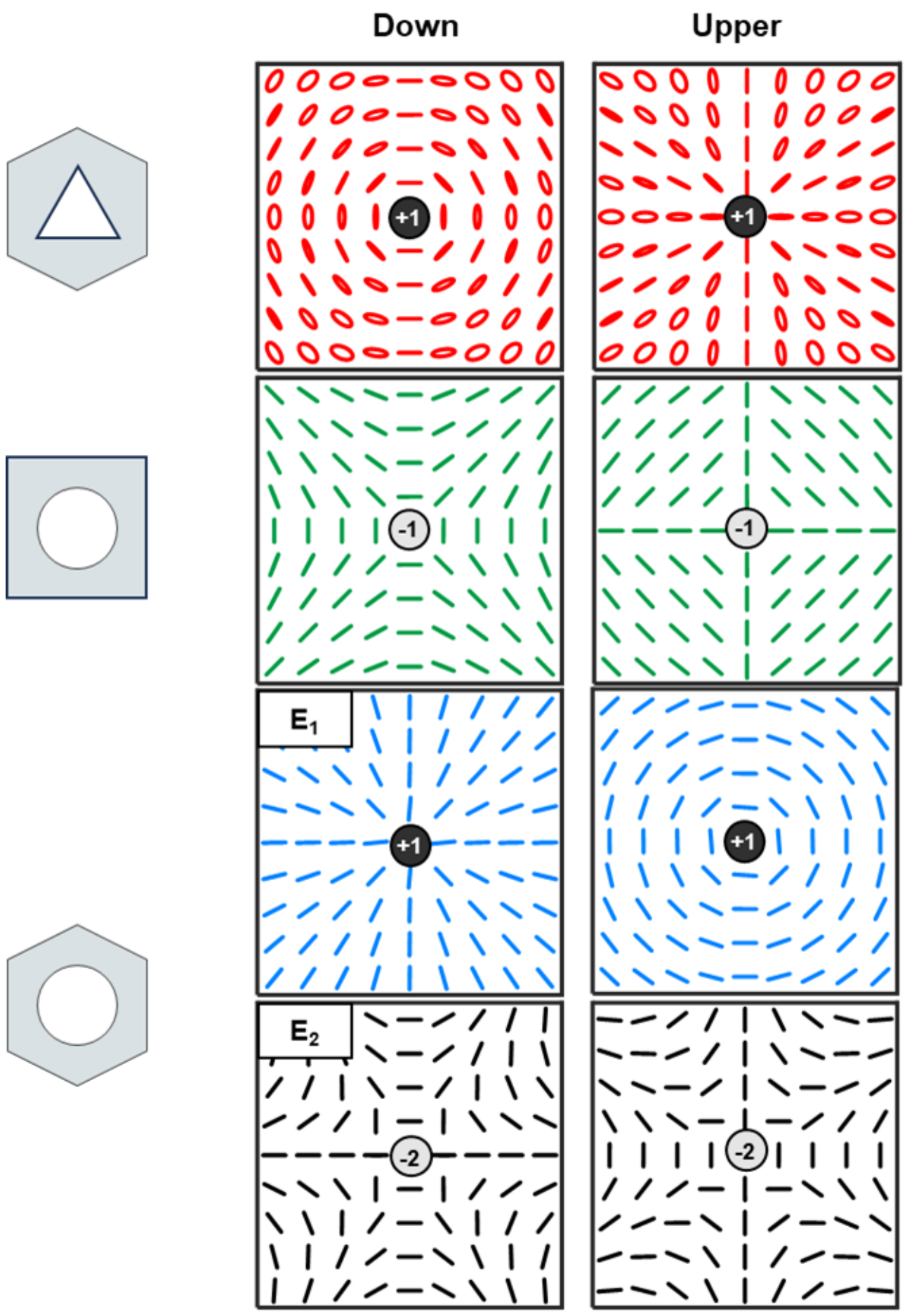

Supplementary Fig. 2. Polarization distribution for all degenerate types in momentum space. The central topological charge matches theoretical predictions. Results correspond to the first TM band for the structures described.

Supplementary Note 2: Evolution of Topological Charge at Degeneracy Points with Symmetry

Compared to singlet bands, the topological charge at degeneracy points is constrained by both symmetry and degeneracy. When high symmetry is reduced to lower symmetry, the mode transitions into corresponding sub-modes according to the compatibility principle, with the topological charge changing as outlined in Table S1. To maintain charge conservation, additional charges must be introduced. The evolution of topological charge in singlet states is relatively straightforward and has been extensively studied. However, degeneracy points involve changes in half-integer topological charges, which require special consideration.

This paper focuses on the E_2 mode of C_{6v} point group, the only degenerate mode that supports BICs, as indicated in Table S1. In our study, the transition of topological charge from − 2 to + 1 generates two additional BICs at the center, rather than simply splitting, as observed in singlet states. When the symmetry is preserved, the combined constraints of the degeneracy point and symmetry fix the charge at -2, regardless of parameter variations. However, once symmetry is broken, the internally accumulated topological charge is released, a unique property of the E_2 mode.

This behavior is not a singular case. As shown in Fig. S3, we calculated the first and second E_2 degeneracy bands in the TE mode and observed the same generation of topological charge. To avoid interference from other bands, these calculations were performed using different structural parameters.

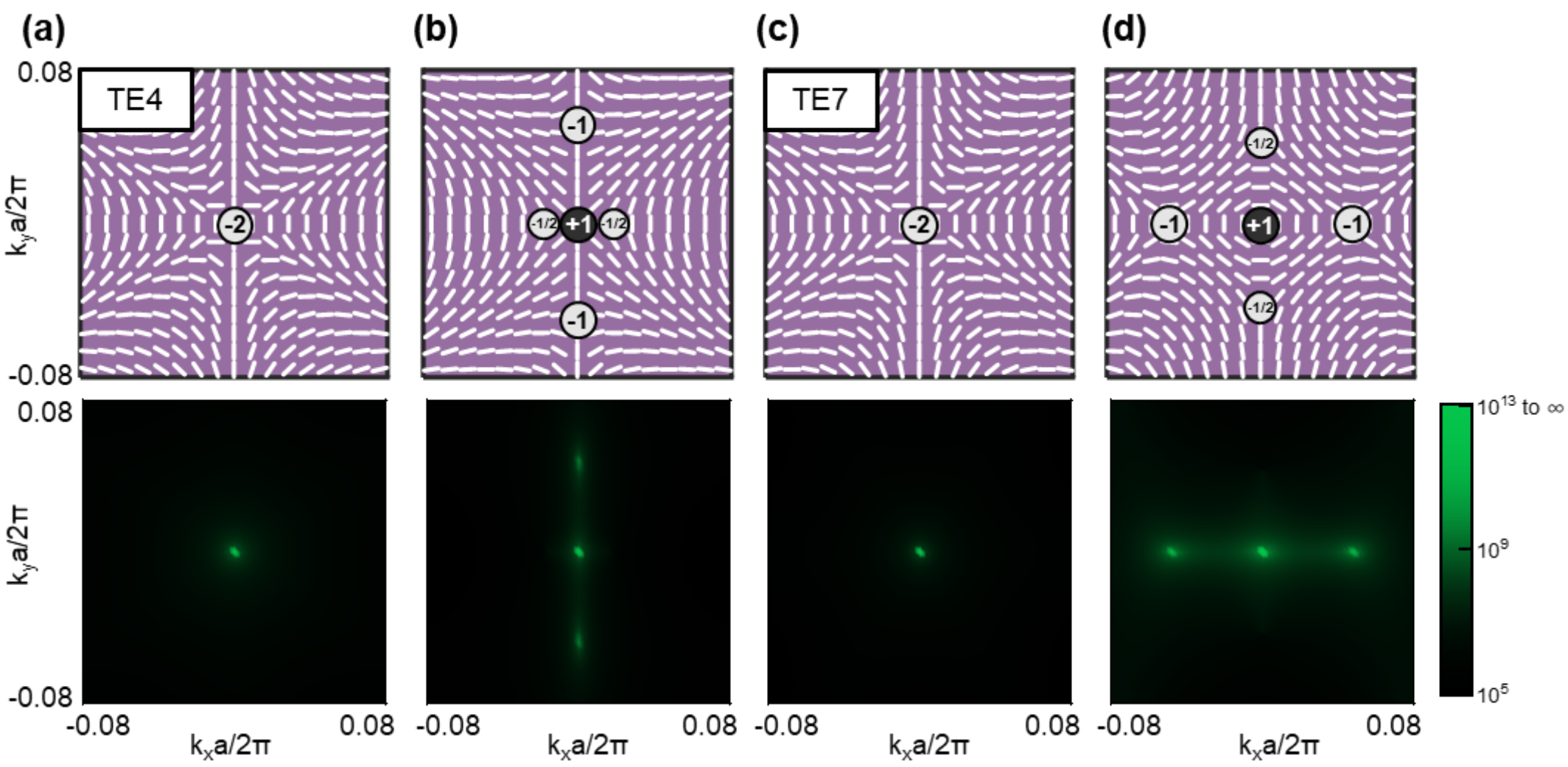

Supplementary Fig. 3. Topological charge splitting at degeneracy points for different bands. Schematic of the evolution of topological charge in momentum space for the first (**a, b**) and second (**c, d**) E_2 degeneracy bands. **a**, The period is *a*, the thickness is *a*, and the air-hole diameter is 0.71*a*. **b**, The thickness is 0.88*a*, and the air-hole diameter is 0.55*a*. Symmetry is broken by converting the circular holes into elliptical ones, following the same procedure as before.

Supplementary Note 3: Evolution of Global Topological Charge with Symmetry

Beyond the degeneracy point itself, changes in the structural symmetry also affect other accidental BICs, mainly by redistributing them in momentum space. Accidental BICs originate from destructive interference of radiative channels associated with Bloch modes and, in principle, can occur at arbitrary locations in the momentum space of any structure. In practice, however, the parameter tuning required to satisfy the cancellation condition is typically stringent; hence they occur more readily in sufficiently symmetric systems and are often pinned to high-symmetry lines. This is because the symmetry along a high-symmetry line naturally enforces a definite polarization (s or p), which greatly reduces the degrees of freedom needed to achieve complete destructive interference among radiation channels. Consequently, topological charges appearing away from high-symmetry lines are generally less robust, or tend to migrate and accumulate onto high-symmetry lines under parameter tuning.

For these reasons, in this work, when the structural symmetry is broken, not only does the degeneracy point release the topological charges, but the surrounding accidental BICs are also rearranged. Figure S4a-k shows the full evolution of the topological charges during symmetry breaking. In the C6-symmetric structure, 12 topological charges are symmetrically distributed along six high-symmetry lines. As the structure is reduced to C2 symmetry, these charges migrate toward the two remaining high-symmetry lines and eventually merge into new charges. For the present parameter configuration, when the charges on the ky axis merged at the center, the charges from the degeneracy point are released simultaneously, yielding three groups of charges merging at the central Γ point, which produces the effect of $Q \propto \mathbf{k}^{-10}$. By controlling the timing of symmetry breaking, different topological-charge configurations can be realized, as shown in Fig. 2a, c in the main text.

Fig.S4l-n shows the evolution of the Q-factor distribution and scaling law along the ky axis as the symmetry-breaking parameter is varied. As the parameter changes, the central Q factor increases continuously and rises more rapidly when the topological charge at the center is about to be released. Ultimately, the scaling behavior near the Γ point evolves from initial $Q \propto \mathbf{k}^{-2}$ to $Q \propto \mathbf{k}^{-10}$.

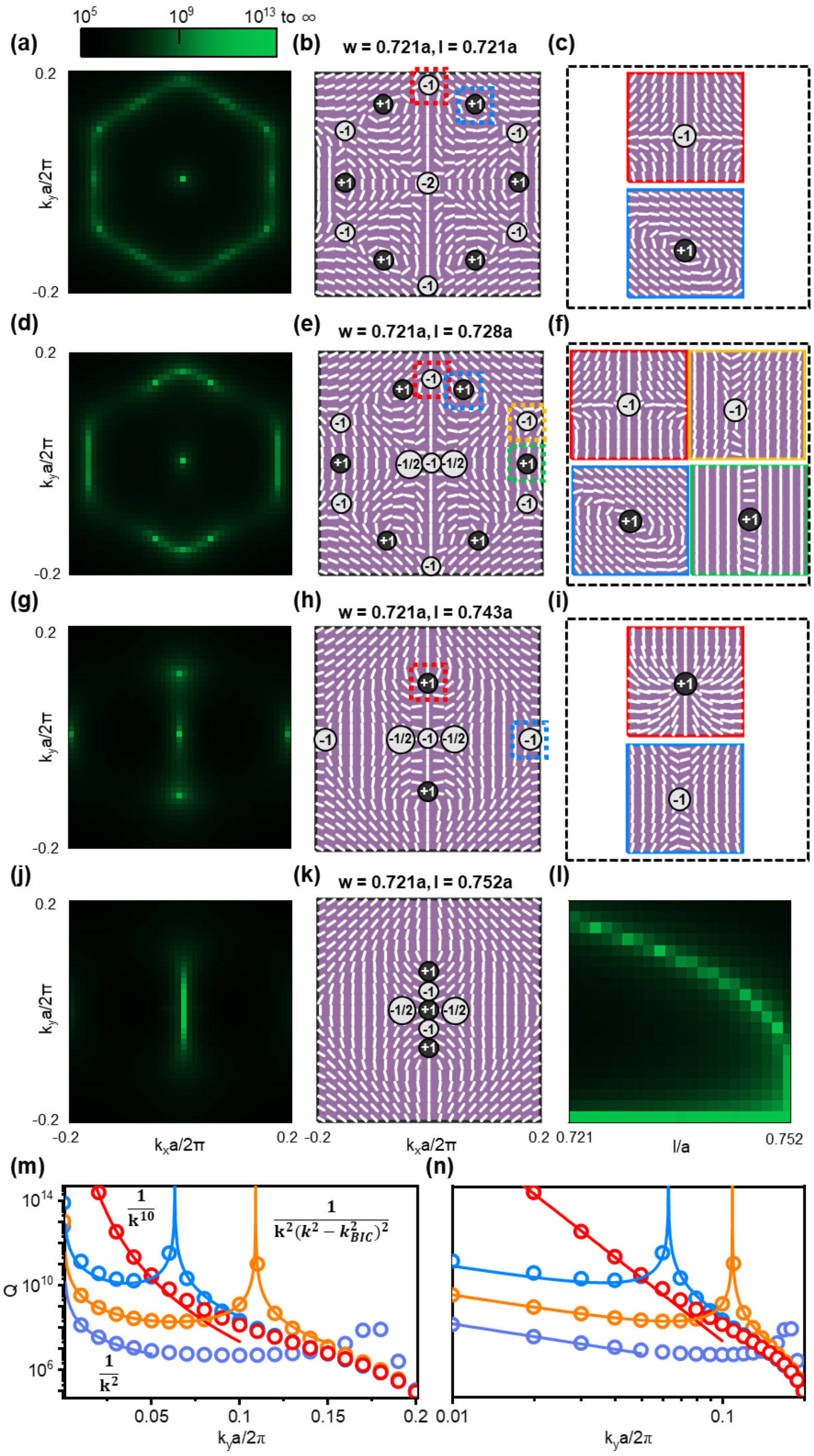

Supplementary Fig. 4. Schematic of the evolution of the global topological charge. Simulated far-field polarization textures and Q-factor maps of the evolution of the outer-ring accidental-BIC topological charges during the symmetry-breaking process. Representative topological charges were selected according to the structural symmetry and enlarged to show their detailed polarization-vortex textures. **a, d, g, j,** Q-factor distribution map during the evolution process. **b, e, h, k**, Far-field polarization textures map during the evolution process. **c, f, i,** Locally enlarged views near the topological charges. The selected topological charges are marked with boxes of the same color. **l,** Variation of the Q-factor distribution along the k_y axis as symmetry is broken. **m, n,** As the structural symmetry is broken, the scaling law of the Q factor changes. The curves in different colors correspond to the different states labeled in l.

Supplementary Note 4: Derivation of Scaling Rule $Q \propto 1/k^{10}$

The far-field polarization field ($\boldsymbol{\varphi}(\mathbf{k}) = c_x(\mathbf{k})\hat{x} + c_y(\mathbf{k})\hat{y}$) is a continuous function of wave vector $\mathbf{k}$. Therefore, the properties of the polarization field can be studied by a Taylor expansion of $c_x(\mathbf{k})$ and $c_y(\mathbf{k})$ near $\mathbf{k} = 0$, as shown below:

$$c_x = \sum a_{mn} k_x^m\, k_y^n, \quad c_y = \sum b_{mn} k_x^m\, k_y^n \qquad (S.8)$$

The polarization configuration in this mode can be obtained by introducing the symmetry of the structure. It can also be derived the scaling rules of Q factor are $Q \propto 1/\mathbf{k}^{2n}$, which n is the central topological charge. However, this rule is not applicable to the degeneracy point BIC. An important reason is that $\mathbf{c}(\mathbf{k})$ is a singularity at $\mathbf{k} = 0$, so it is impossible to expand into Taylor series.

From the perspective of topological charge, the additional vortex caused by the degeneracy point does not contribute to the enhancement of the Q factor. Therefore, more strictly, n in the scaling rule $Q \propto 1/\mathbf{k}^{2n}$ should be the effective charge excluding the vortex brought by the band structure. In this paper, the effective topological charge of degenerate BIC should be – 1. So, it will satisfy the $Q \propto 1/\mathbf{k}^2$.

When the structural symmetry breaking causing the degeneracy at Γ point is splitting, we can continue to use Eq. (S.8) to study the polarization field. The lifting of the degeneracy point simplifies our analysis: following a procedure analogous to the conventional derivation, we can obtain the final result, with the only modification being a more elaborate expansion to account for the additional set of topological charges. We consider the A_1 representation of C_{2v} point group. Intruding the intrinsic mirror symmetry $\sigma_x \boldsymbol{\varphi}(\mathbf{k}) = \boldsymbol{\varphi}(\sigma_x \mathbf{k})$ and $\sigma_y \boldsymbol{\varphi}(\mathbf{k}) = \boldsymbol{\varphi}(\sigma_y \mathbf{k})$, we have

$$c_x = a_1 k_x + a_2 k_x k_y^2 + a_3 k_x^3 + \cdots \qquad (S.9)$$

$$c_y = b_1 k_y + b_2 k_y k_x^2 + b_3 k_y^3 + \cdots \qquad (S.10)$$

These equations also satisfy the rotational symmetry $C_2 \boldsymbol{\varphi}(\mathbf{k}) = \boldsymbol{\varphi}(C_2 \mathbf{k})$. When only a central isolated BIC is present, the scaling law is $Q \propto 1/|\boldsymbol{\varphi}(\mathbf{k})|^2 \propto 1/(k_x + r k_y)^2$ by keeping the first order of expansion. As a result, the iso-Q contour is an ellipse with different attenuation coefficients on the k_x and k_y axes. As studied in this paper, the BIC of C2v system usually appears on the symmetry axis. Let $k_x = 0$, we study the properties on the k_y axis:

$$c_x = 0, \qquad c_y = b_1 k_y + b_3 k_y^3 + b_5 k_y^5 + \cdots \qquad (S.11)$$

When an additional BIC appears along the k_y axis, an additional constraint

$c_y(0, k_{BIC}) = k_{BIC}(b_1 + b_3 k_{BIC}^2) = 0$ is introduced, simplifying Eq. (S.11) to:

$$c_y = b_3 k_y \left(k_y^2 - k_{BIC}^2\right) \quad (S.12)$$

Thus, the scaling law becomes $Q \propto 1/[k_y\left(k_y^2 - k_{BIC}^2\right)]^2$, When two additional BICs are present, as in this study, two sets of constraints are required: $c_y(0, k_{BIC1}) = 0$, and $c_y(0, k_{BIC2}) = 0$. Substituting these into the expansion yields:

$$\begin{cases} b_1 = b_5 k_{BIC1}^2 k_{BIC2}^2 \\ b_3 = -b_5 (k_{BIC1}^2 - k_{BIC2}^2) \end{cases} \quad (S.13)$$

$$c_y = b_5 k_y \left(k_y^2 - k_{BIC1}^2\right)\left(k_y^2 - k_{BIC2}^2\right) \quad (S.14)$$

Therefore, the scaling law becomes $Q \propto 1/[k_y\left(k_y^2 - k_{BIC1}^2\right)\left(k_y^2 - k_{BIC2}^2\right)]^2$. When k_{BIC1} and k_{BIC2} both approach zero, this approximation leads to $Q \propto 1/k_y^{10}$. This Q-factor distribution represents the highest achievable under current theoretical models, exploiting the unique properties of degenerate BICs. By using symmetry breaking to release internal topological charges, we achieve the aggregation of multiple off-Γ BICs. Furthermore, when more groups of topological charges are involved, additional terms must be retained in the derivation.

Supplementary Note 5: Typical Paradigms of Topological Charge Manipulation

This work demonstrates how concentrating all topological charges near the central Γ point improves the robustness of the Q factor. However, by controlling the timing of degeneracy point breaking, it is possible to achieve flexible manipulation of these topological charges. Several typical manipulation paradigms are shown in Fig. S5.

In Fig. S5a, the interval arrangement of three types of topological charges is displayed, resulting in five BIC points along the k_y-axis. This configuration is valuable for designing multi-angle devices. Figs. S5b and S5c show that two BICs can merge at arbitrary locations while preserving the central BIC, meaning no trade-off is necessary to enhance the Q factor at off-Γ points. In Fig. S5d, after rotating the air holes to break the mirror symmetry of the structure, it is shown that this series of manipulations can occur at any position in momentum space. All structural parameters are the same as those in Fig. 2, except for changes in w and l.

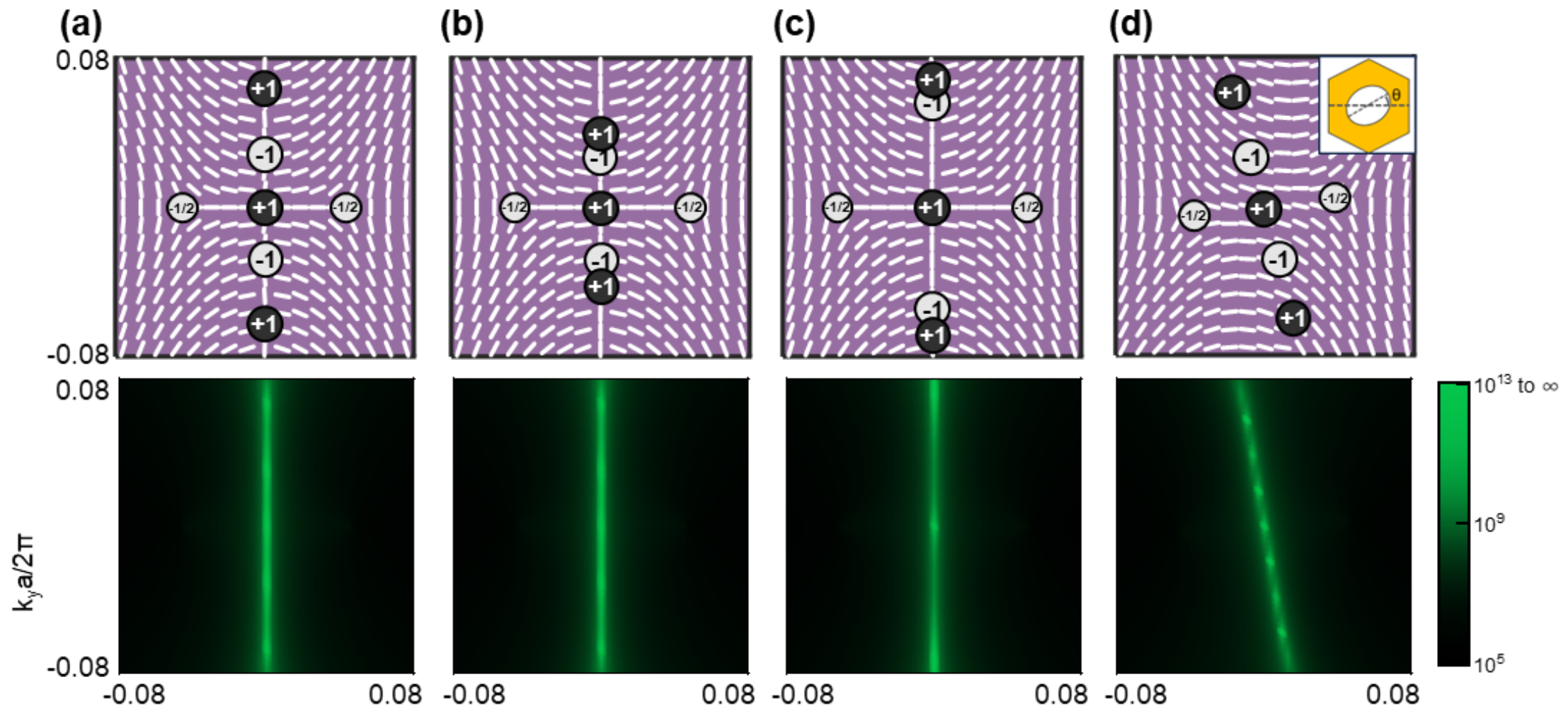

Supplementary Fig. 5. Further manipulation of degenerate BICs. a, Five separated BICs along the k_y-axis with w=0.703a and l=0.743a. **b, c**, Merging of two BICs at different points with w=0.703a, l=0.704a and w=0.688a, l=0.739a. **d**, A series of off-axis BICs with w=0.697a, l=0.739a, θ=4°.

Supplementary Note 6: Details About Two Comparison Samples

To demonstrate the superiority of our proposed design, we constructed two additional sample types, each conforming to the scaling laws $Q \propto \mathbf{k}^{-2}$ and $Q \propto \mathbf{k}^{-6}$, respectively, under the same band. The schematic designs of these two samples in momentum space are shown in Fig. S6. Experimental results indicate that the Q-factors for these two designs are significantly lower than those achieved with our degeneracy-breaking design which follows scaling laws $Q \propto \mathbf{k}^{-10}$, as shown in Fig. S6. It is important to note that the periodicities of the three sample types differ due to the measurement system's wavelength range limitations. The additional inset in Fig. S6d shows the fine polarization-vortex structures of the two types of topological charges under a slight parameter variation, corroborating the charge assignments labeled in the figure.

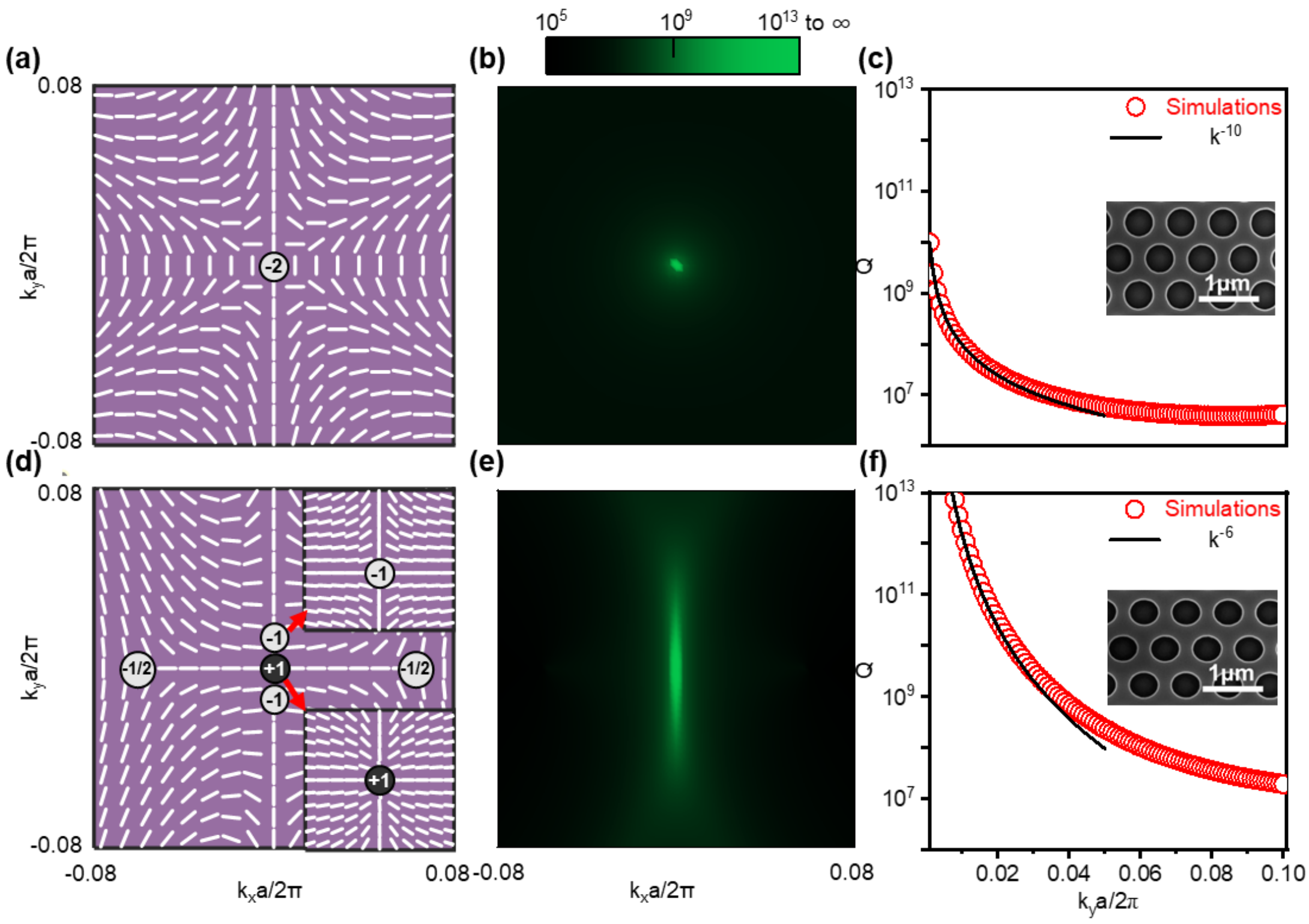

Supplementary Fig. 6. Isolated-BIC and merging-BIC designs. a, b, d, e, Schematics of the far-field polarization vector and Q-factor distributions in momentum space for the two types of samples. The two insets in d display the polarization-vector configurations of the central and upper topological charges under slight parameter variations. **c, f**, Simulated Q-factors (circular lines) and their corresponding fitted curves (solid lines). Insets show SEM images of the fabricated samples.

Supplementary Note 7: Robustness of Device Design and Fabrication

1. Influence of Substrate on Robustness:

Generally, achieving flawless BICs requires up-down mirror symmetry. Introducing a substrate can increase mode leakage and reduce the Q factor. Before etching the oxide layer, we performed preliminary characterizations of samples with the substrate. The typical band structure is illustrated by the isofrequency slice in Fig. S7. Despite the substrate, all five samples maintained Q factors above 10^5, with the highest reaching 3×10^5. An additional sample was included due to constraints in the laser's wavelength range. After etching away the oxide layer, one sample exceeded the measurement range. These results indicate that our design can retain high Q factors even with the substrate present, easing the fabrication process.

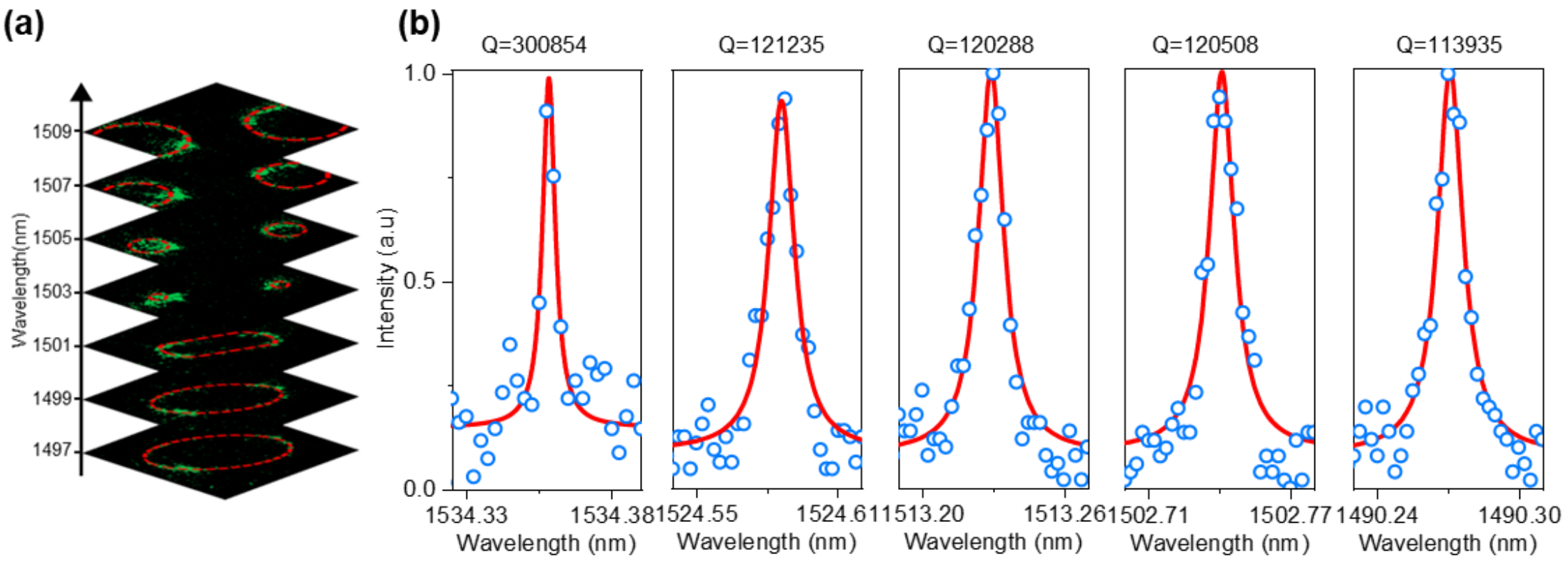

Supplementary Fig. 7. Experimental results for samples with a substrate. a, Isofrequency contours of a sample measured at seven selected wavelengths. **b**, Scattering spectra of five samples near the Γ point, all showing Q factors above 10^5.

2. Tolerance to Fabrication Errors in Topological Design:

In practice, achieving an infinite Q factor or a $Q \propto \mathbf{k}^{-10}$ distribution is challenging due to fabrication errors. To simulate these deviations, we modeled a 4×4 supercell with random offsets in the diameter of the air holes' short axis ([−5nm,5nm]) and proportional adjustments to the long axis. Additionally, we introduced central displacements of [−3nm,3nm] in the x and y axes for each hole. As shown in Fig. S8, our design maintains high Q factors across a wide range of momentum space, despite these errors. Compared to traditional designs, our design performs better in terms of Q factor and attenuation stability. Although increased fabrication errors lead to a slight decline in Q factor, high levels are still retained, and the attenuation curve remains stable.

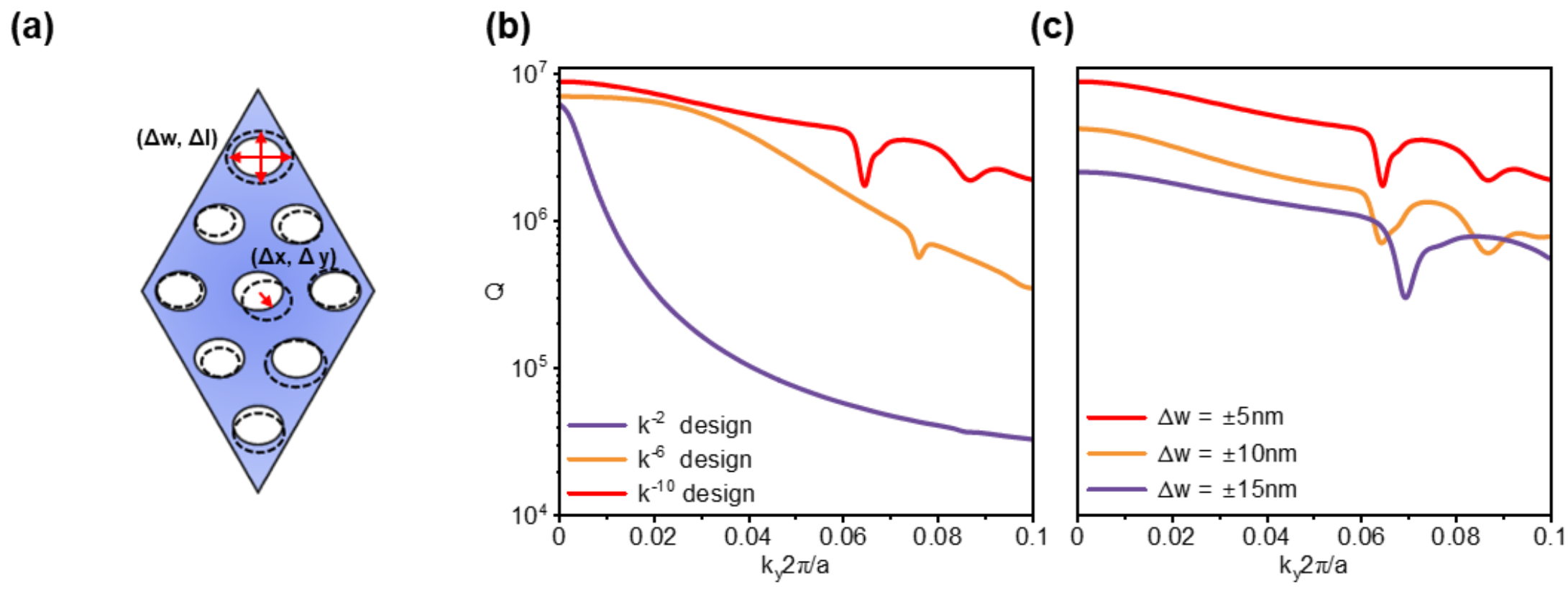

Supplementary Fig. 8. Robustness under fabricating deviation. a, Schematic of actual fabricated samples, with various error (dash line) in the size and position of air holes. **b**, The Q factor distribution of deviation introduced PhCs based on three different design. In the simulations, hole size deviation Δ*w*=[-5nm,5nm], Δ*l* varies proportionally. Central shift Δ*x*=Δ*y*=[-3nm,3nm]. **c**, The Q factor distribution with a higher fabricated error *Δw*=±5 to ±15nm.

3. Impact of Wafer Thickness Deviation on Robustness:

Since the thickness of the sample differs from standard commercial SOI top silicon, deviations in thickness are inevitable. We calculated the impact of slab thickness variations from 418 nm to 442 nm, considering fabrication errors similar to those in Fig. S8. As shown in Fig. S9, our design maintains high Q factors across a wide range of thicknesses. Minor deviations in thickness have minimal impact near the Γ-point. Noticeable differences only arise for Q factors far from the Γ-point as the thickness deviation increases, resulting in a slight acceleration of Q factor attenuation with increasing wave vector (k). These results demonstrate the robustness of our design to complex fabrication errors, confirming its resilience to various deviations.

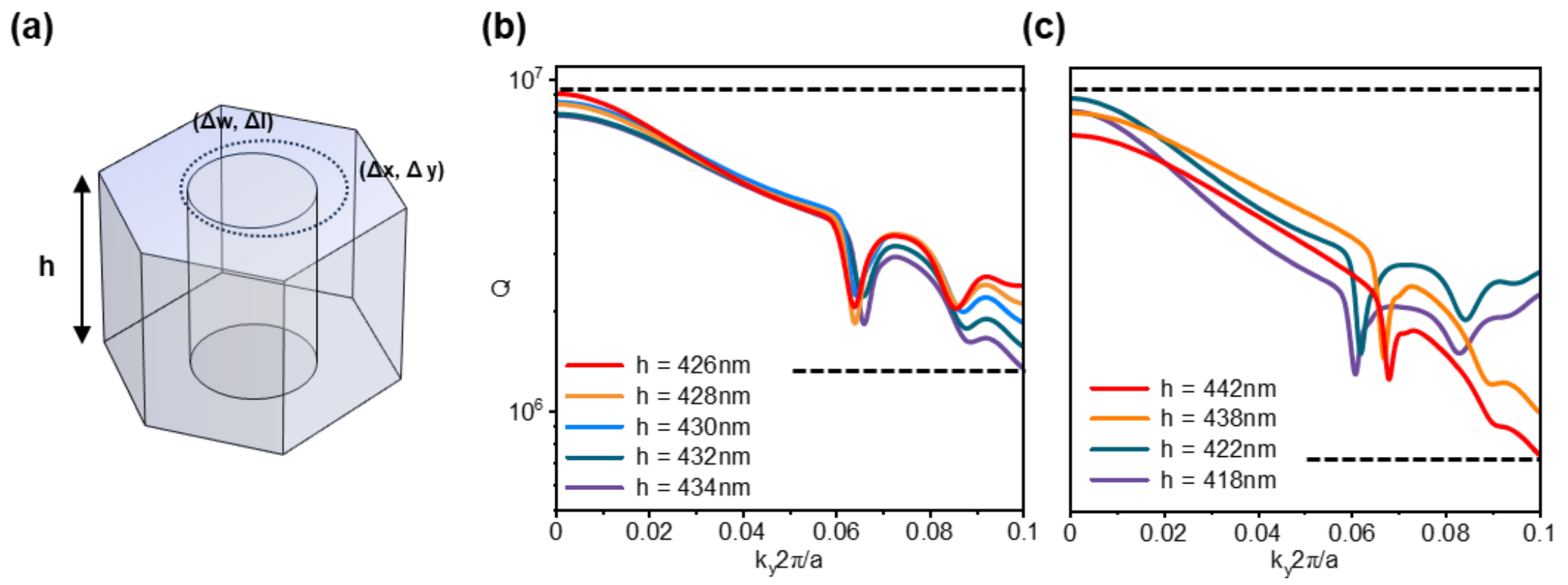

Supplementary Fig. 9. Influence of structural thickness on the Q factor. **a**, Schematic of thickness deviation and other fabrication errors. **b, c**, Q factor distribution with thickness deviations. Small deviations primarily affect regions far from the Γ-point, while larger deviations lead to more pronounced attenuation of the Q factor.

Supplementary Note 8: Construction of the spectroscopic sensing array

Guided by our theoretical framework, we built a spectroscopic sensing array that incorporates one hundred metasurface. The metasurface array used for data acquisition included slight variations in lattice periodicity and elliptical short-axis lengths, while maintaining a constant device-layer thickness and elliptical aspect ratio $l/w = 1.044$. The central parameters are set to lattice periodicity a = 677 nm, thickness h = 448nm, elliptical short-axis lengths w = 478.2nm. Fig. S10 presents the spectrometer array layout along with its scanning-electron-microscope (SEM) image.

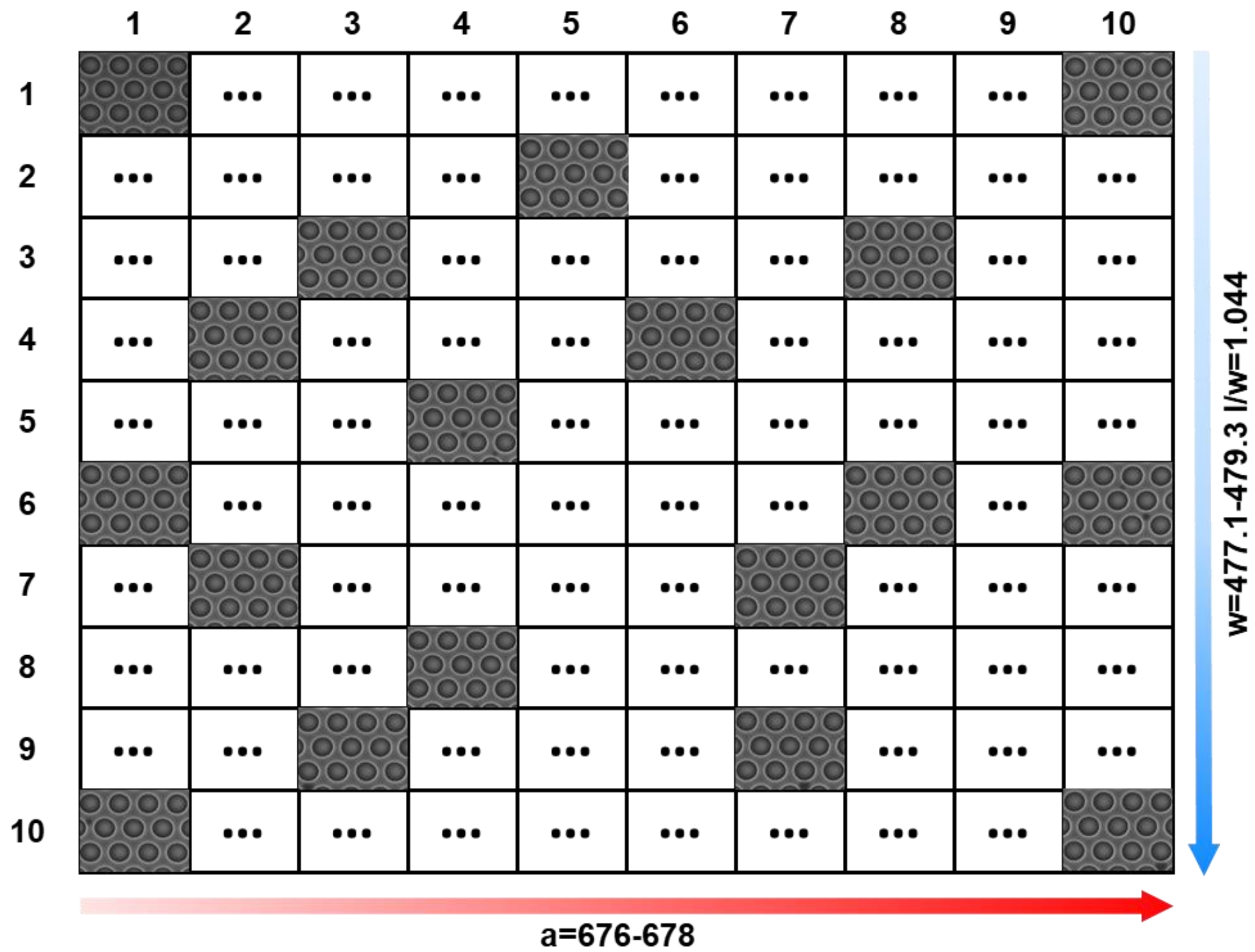

Supplementary Fig. 10. Spectrometer-array architecture and SEM image of a single metasurface unit. These metasurfaces were fabricated with slight variations in lattice periodicity (676–678 nm) and elliptical short-axis length (477.1–479.3 nm), while maintaining a constant device-layer thickness of 448.5 nm and an elliptical aspect ratio of l/w=1.044.

The spectral resolution of a miniaturized spectrometer is fundamentally determined by the ultra-narrow resonant response supported by each constituent unit cell. Fig.S11a compiles the resonance characteristics of all fabricated samples, while Fig.S11b, c magnify the line shapes of several representative spectra. The spectra were acquired using the same measurement procedure as in our previous experiments. In contrast to the previous Q-factor measurements, the k-space-selective pinhole was opened during spectrometer acquisition, enabling broadband resonance signal collection. Our platform

supporting ultrahigh Q resonances over an extended region of momentum space, so the recorded emission manifests as a densely packed comb of ultranarrow peaks. This information-rich spectral pattern provides a solid foundation for accurate spectrum reconstruction.

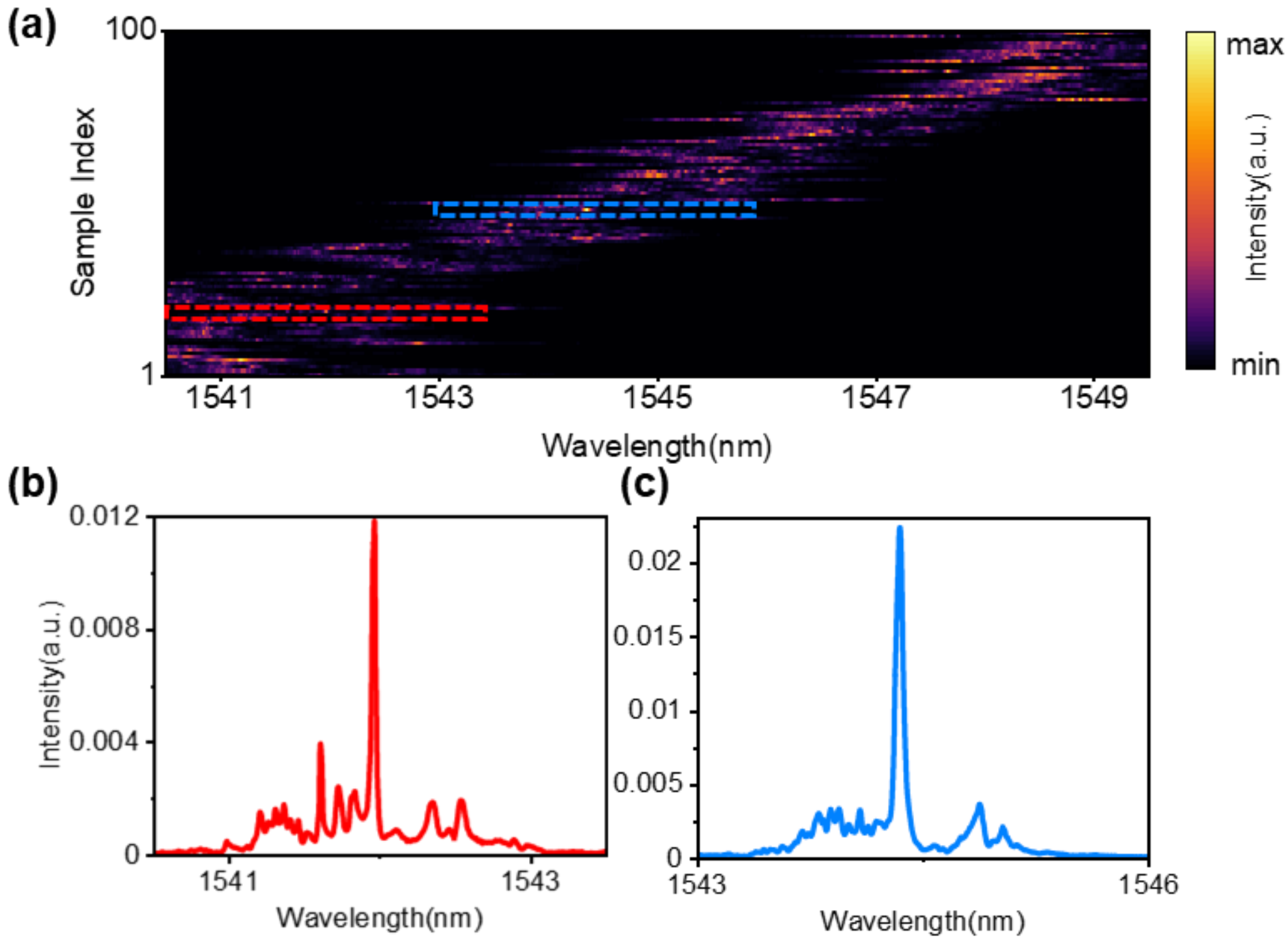

Supplementary Fig. 11 Schematic of the spectral response of the array. a, The response matrix corresponding to the spectral array composed of 100 samples. **b,** The specific scattering spectrum of the sample labeled in a.

Supplementary Note 9: Performance comparison for reported miniaturized spectrometers

Miniaturized spectrometers can be broadly classified by the way light is introduced into the device: (1) free-space-coupled and (2) on-chip-coupled. Free-space-coupled architectures-such as those based on metasurfaces-enable direct detection. However, for high-sensitivity or high-precision measurements their performance is ultimately limited by radiation leakage from openness. A common workaround is to employ closed resonant cavities-e.g., waveguides or microring resonators-whose suppressed out-of-plane loss can deliver much higher spectral resolution. The price is additional complexity: light must be routed into, through, and out of the chip with dedicated coupling and bus-waveguide structures, which complicate the setup and hinder practical deployment in compact sensing platforms. **Table S2** summarizes the coupling configurations and key performance metrics of the latest miniaturized spectrometers reported. Several representative commercial instruments are listed for comparison.

Table S2. Performance for reported miniaturized spectrometers

Fiber-to-Chip/Free space	Resolution (nm)	Typical Wavelength (nm)	Ref
**Fiber-to-Chip**	0.3	770	1
	0.047	1550	2
	0.2	1550	3
	0.47	1571	4
	0.01	1510	5
	0.005	1550	6
	0.01	1520	7
	0.005	1550	8
	0.4	1550	9
	0.008	1550	10
	2.45	650	11
**Free space**	2	635	12
	10	650	13
	1.2	650	14
	1.7	550	15
	10	1000	16
	~15	6475	17
	3	625	18
	**0.02**	**1545**	**Our work**
Shamrock 750	0.09	1550	19
iHR550	0.07	1550	20

Supplementary References

1. Hartmann W, Varytis P, Gehring H, Walter N, Beutel F, Busch K, et al. Waveguide-Integrated Broadband Spectrometer Based on Tailored Disorder. Adv Opt Mater 2020, 8(6): 1901602.
2. Zhang Z, Song Q, Xiao S, Xu K. Single-Shot on-Chip Diffractive Speckle Spectrometer with High Spectral Channel Density. Laser & Photonics Reviews 2025, 19(9): 2401987.
3. Sun C, Chen Z, Ye Y, Weng Y, Lei K, Ma H, et al. Integrated Microring Spectrometer with In-Hardware Compressed Sensing to Break the Resolution-Bandwidth Limit for General Continuous Spectrum Analysis. Laser & Photonics Reviews 2023, 17(11): 2300291.
4. Zheng SN, Zou J, Cai H, Song JF, Chin LK, Liu PY, et al. Microring resonator-assisted Fourier transform spectrometer with enhanced resolution and large bandwidth in single chip solution. Nature Communications 2019, 10(1): 2349.
5. Yao C, Xu K, Zhang W, Chen M, Cheng Q, Penty R. Integrated reconstructive spectrometer with programmable photonic circuits. Nature Communications 2023, 14(1): 6376.
6. Zhang L, Zhang M, Chen T, Liu D, Hong S, Dai D. Ultrahigh-resolution on-chip spectrometer with silicon photonic resonators. Opto-Electronic Advances 2022, 5(7): 210100-210101.
7. Redding B, Fatt Liew S, Bromberg Y, Sarma R, Cao H. Evanescently coupled multimode spiral spectrometer. Optica 2016, 3(9): 956-962.
8. Xu H, Qin Y, Hu G, Tsang HK. Cavity-enhanced scalable integrated temporal random-speckle spectrometry. Optica 2023, 10(9): 1177-1188.
9. Zhao Y, Guo X, Xiang J, Zhao Z, Zhang Y, Xiao X, et al. Miniaturized computational spectrometer based on two-photon absorption. Optica 2024, 11(3): 399-402.
10. Zhang YJ, Albrow-Owen T, Zhao ZY, Chen YP, Zhao YT, Joyce H, et al. Miniaturized disordered photonic molecule spectrometer. Light-Sci Appl 2025, 14(1): 144.
11. Cui XQ, Nigmatulin F, Wang L, Reduto I, Liapis AC, Du MD, et al. Miniaturized spectral sensing with a tunable optoelectronic interface. Science Advances 2025, 11(4): eado6886.
12. Li X, Wang J, Yu F, Chen J, Chen X, Lu W, et al. Nonlinear memristive computational spectrometer. Light: Science & Applications 2025, 14(1): 47.
13. Wang J, Pan B, Wang Z, Zhang J, Zhou Z, Yao L, et al. Single-pixel p-graded-n junction spectrometers. Nature Communications 2024, 15(1): 1773.
14. Du X, Wang Y, Cui Y, Rao G, Huang J, Chen X, et al. A microspectrometer with dual-signal spectral reconstruction. Nature Electronics 2024, 7(11): 984-990.
15. Tang F, Wu JJ, Albrow-Owen T, Cui HX, Chen FJ, Shi YQ, et al. Metasurface spectrometers beyond resolution-sensitivity constraints. Science Advances 2024, 10(49): eadr7155.
16. Zhang GY, Albrow-Owen T, Peng WJ, Liang X, Zhang XM, Wang P, et al. Stress-engineered ultra-broadband spectrometers. Science Advances 2025, 11(20): eadu4225.
17. Tittl A, Leitis A, Liu MK, Yesilkoy F, Choi DY, Neshev DN, et al. Imaging-based molecular barcoding with pixelated dielectric metasurfaces. Science 2018, 360(6393): 1105-1109.
18. Yoon HH, Fernandez HA, Nigmatulin F, Cai WW, Vane ZY, Cui HX, et al. Miniaturized spectrometers with a tunable van der Waals junction. Science 2022, 378(6617): 296-299.
19. Instrmnt, O. https://andor.oxinst.com，(accessed 18 Jan 2026).
20. Horiba. https://www.horiba.com/，(accessed 18 Jan 2026).
