# Supplementary material for: High-order photonic degeneracy enables topological reconfiguration in bound states in the continuum
Source: Nat Commun. 2026 Jul 22;17:9203. doi: 10.1038/s41467-026-75967-w (PMC13522528; doi:10.1038/s41467-026-75967-w)
Supplement: Supplementary file 2 [file 41467_2026_75967_MOESM2_ESM.pdf]

nature portfolio

Peer Review File

High-Order Photonic Degeneracy Enables Topological Reconfiguration in Bound States in the Continuum

Corresponding Author: Professor Guanhai Li

This file contains all reviewer reports in order by version, followed by all author rebuttals in order by version.

Attachments originally included by the reviewers as part of their assessment can be found at the end of this file.

Version 0:

Reviewer comments:

Reviewer #1

(Remarks to the Author)
None.

Reviewer #2

(Remarks to the Author)
Fu et al. demonstrated a BIC with a Q \prop k^-10 scaling law in a single-layer all-dielectric metasurface, and experimentally realized free-space Q-factors above 1x10^6 in the near-infrared regime. These performances are strikingly high compared with existing reports in the literature. They also demonstrated picometer-scale spectral discrimination making use of a 100-pixel metasurface array with such ultranarrow resonance. The results are very interesting and would be of great importance in the field of metasurfaces. However, before this manuscript can be considered for publication, the authors should carefully address the following problems.

1. (Major) On the novelty. At first glance, the concept and the structure is very similar to Kang et al. Light: Sci. Appl. 11, 228 (2022), which, unfortunately, was not cited by the authors. In that paper, photonic crystal slabs with a triangular lattice of cylindrical air holes were also adopted, and merging multiple BICs with a higher-order BIC (of higher-order topological charges) also greatly improves the Q factor with a k^-8 scaling law. The authors should comment that paper properly and clarify in details why Q~k^-10 can be obtained in this work but only k^-8 was obtained in that work. Accordingly, the novelty of this work should be rephrased since previously the authors only considered k^-6 as the upper limit in the literature.

2. (Major) The authors should explicitly clarify how topological charge conservation is manifested during symmetry breaking and charge inversion.

3. (Major) In Fig. 4df, it is clear that Q factors for both k^-6 and k^-10 drop as ky*2*pi/a is smaller than 0.01 (approaching the Gamma point), but increase for k^-2. Why this happens?

4. (Major) On the fabrication tolerance. In the Abstract, “free-space Q-factors above 1.1x10^6 together with +-3% geometric robustness” is overclaimed. According to this claim, the readers may expect Q factors above 1.1x10^6 for any geometric variations within +-3%. However, in Fig. 4d, only the short-axis width has 3% variation (not +-3%, which is 6% indeed), and all the samples (especially the red dots) have measured Q factors above 4x10^5, not 1.1x10^6.

5. (Major) The authors fabricated samples based on a silicon-on-insulator wafer. They etched the oxide layer in order to ensure up-down mirror symmetry. However, the bottom silicon reflects strongly and degrade the transmission spectra, especially on the transmission efficiency when used for miniaturized spectrometers, although the Q factors are not changed. In this work, all the intensities in the measured spectra adopt arbitrary unit (a.u.), what are the original experimental data? Could the authors provide a few in the SM? Additionally, could the authors compare the simulated transmission spectra with and without the bottom silicon layer when the oxide layer is etched or not?

6. (Minor) In Fig. 2c, the half-integer topological charges should be more clearly indicated to improve clarity and reader

comprehension.

Reviewer #3

(Remarks to the Author)
Please see the attach file: NCOMMS-26-005383-Comments.docx.

Version 1:

Reviewer comments:

Reviewer #2

(Remarks to the Author)
The authors have satisfactorily addressed all the concerns raised by all the reviewers. Therefore, I'd like to recommend acceptance of the revised manuscript.

Reviewer #3

(Remarks to the Author)
The authors have addressed all our concerns, so I recommend the publication of the manuscript in its present version.

Open Access This Peer Review File is licensed under a Creative Commons Attribution 4.0 International License, which permits use, sharing, adaptation, distribution and reproduction in any medium or format, as long as you give appropriate credit to the original author(s) and the source, provide a link to the Creative Commons license, and indicate if changes were made.
In cases where reviewers are anonymous, credit should be given to 'Anonymous Referee' and the source.
The images or other third party material in this Peer Review File are included in the article's Creative Commons license, unless indicated otherwise in a credit line to the material. If material is not included in the article's Creative Commons license and your intended use is not permitted by statutory regulation or exceeds the permitted use, you will need to obtain permission directly from the copyright holder.
To view a copy of this license, visit https://creativecommons.org/licenses/by/4.0/

Response to Reviewer 1

Comment 1: In Figure 2c, the topological charges should sum to -2 to obey charge conservation. However, if you sum the charges, you get a charge of 0.

Reply: We thank the Reviewer for this comment. We fully agree that topological charge conservation must be strictly satisfied throughout the evolution, and we clarify that no violation of charge conservation occurs in our system. The apparent discrepancy in Fig. 2c arises from an ambiguity in the original presentation, rather than from the underlying physics.

Specifically, Figs. 2a-c were originally intended to illustrate the combined outcome of two coupled but physically distinct processes: (i) the evolution of the outer-ring accidental BIC branch, and (ii) the release and redistribution of topological charges from the lifted degeneracy at Γ.

In Fig. 2c, the two +1 charges along the ky axis originate from the outer-ring accidental BICs, whereas the central integer and half-integer charges arise from the degeneracy-induced charge reservoir. After the structural symmetry is broken, the accidental BICs on the outer ring concentrate onto the new symmetry axis, forming two +1 topological charges on the ky axis. These two contributions were presented simultaneously in a single panel, which can give the impression that the total charge does not sum to -2 when visually counted. However, when the full evolution is tracked continuously, the global topological charge is conserved at every stage, and the apparent imbalance is resolved.

To eliminate this ambiguity, we have restructured the presentation of the topological evolution in the revised manuscript. In particular, we now explicitly separate and continuously track the evolution of the outer accidental-BIC branch and the degeneracy-released charges, and provide a complete momentum-space evolution map in the Supplementary Note 3. This reconstruction clearly shows the processes of charge release, inversion, and merging, and makes the charge conservation unambiguous. In addition, we have added detailed annotations to Fig. 2a–c, as shown in Fig. R1, and

expanded the corresponding discussion in the main text ((highlighted in red font in lines 155-157 and lines161-178).

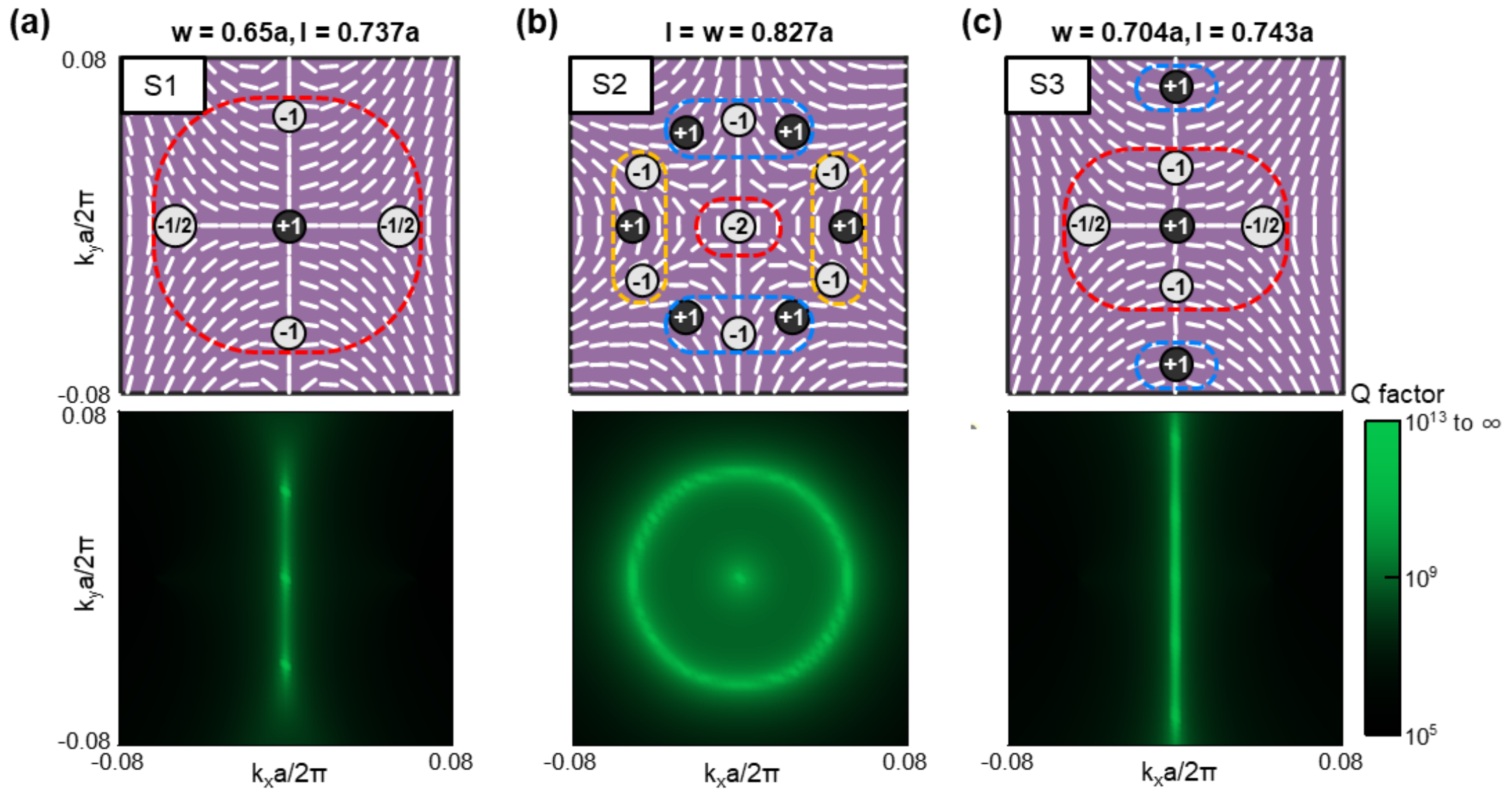

Figure R1 a–c, Simulated far-field polarization textures and Q-factor maps of the TM_3 band under three representative unit-cell configurations. The dashed boxes in three different colors mark the evolution of different groups of topological charges under symmetry breaking. **a**, Symmetry breaking lifts the E_2 degeneracy and releases multiple topological charges, including ±1 and ±1/2 vortices. **b**, In the unperturbed C_6-symmetric structure, a central charge of –2 appears at Γ, corresponding to a symmetry-protected BIC. After the symmetry is reduced to C_2, the 12 topological charges originally distributed along the six symmetry axes will concentrate onto the two remaining symmetry axes and evolve into four topological charges. **c**, Combining symmetry breaking and accidental BIC engineering leads to three distinct topological charges aligned along the k_y-axis.

Comment 2: Even further, there are some discrepancies in the major axis of the polarization ellipses some of the charges in Figure 2b, don’t display the correct winding behavior for the assigned topological chargers.

Reply: We thank the Reviewer for this careful observation. We would like to clarify that the assigned topological charges in Fig. 2b are physically correct, and that the inability to clearly visualize the winding behavior arise from limitations in the visual representation of the polarization field, rather than from incorrect charge identification.

In the original figure, the polarization map was plotted with a sampling density

chosen to preserve the global polarization distribution across the entire Brillouin zone. While this provides a clear overview of the overall charge landscape, it inevitably reduces the resolution of the local polarization vortices, making the winding behavior of individual charges-especially closely spaced or weakly separated ones -less visually distinguishable. This can give the impression that some polarization ellipses do not follow the expected rotation.

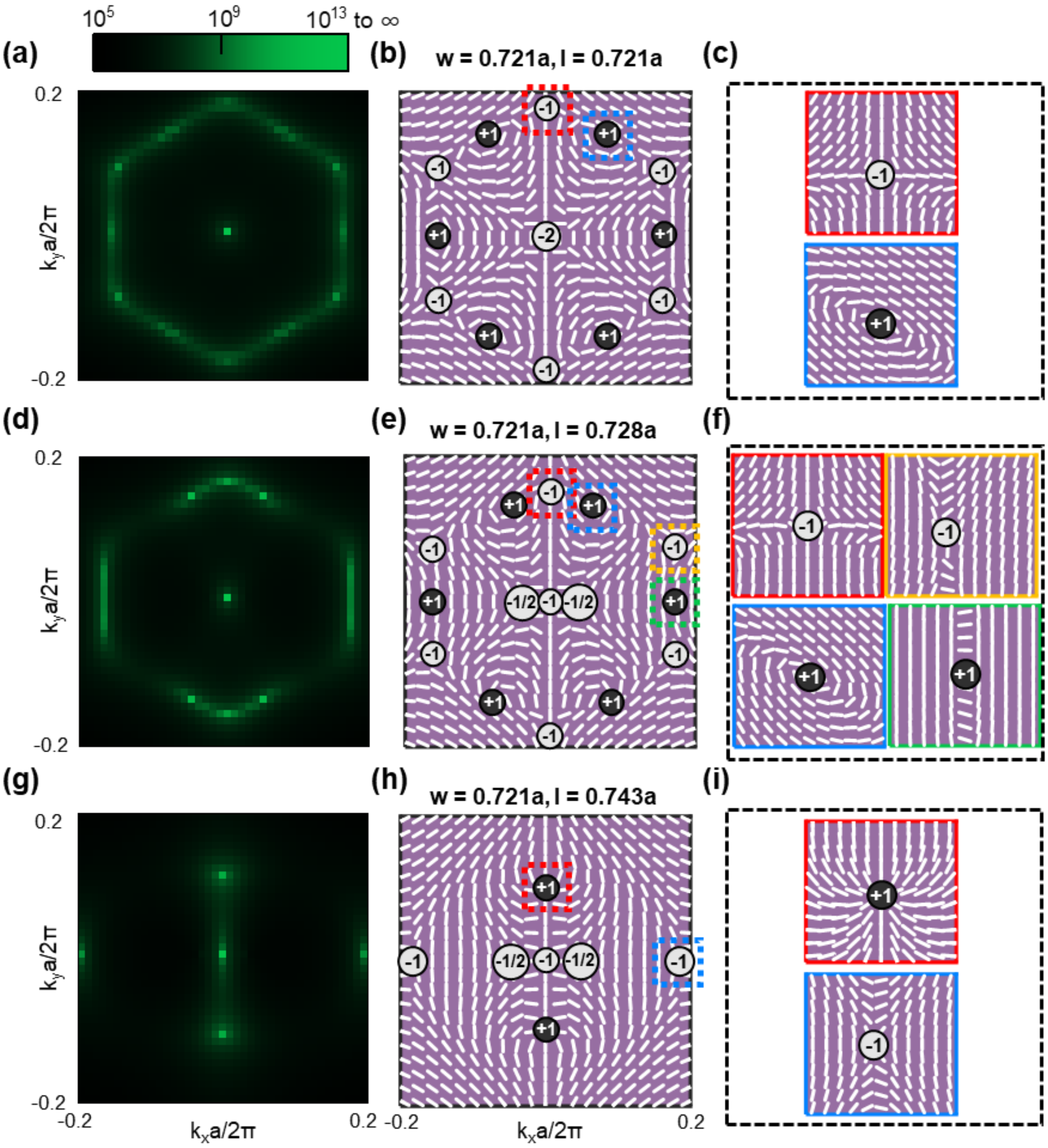

Figure R2 Simulated far-field polarization textures and Q-factor maps of the evolution of the outer-ring accidental-BIC topological charges during the symmetry-breaking process. Representative topological charges were selected according to the structural symmetry and enlarged to show their detailed polarization-vortex textures. **a, d, g**, Q-factor distribution map during the evolution process. **b, e, h**, Far-field polarization textures map during the evolution process. **c, f, i**, Locally enlarged views near the topological charges. The selected topological charges are marked with boxes of the same color.

To address this issue and eliminate other potential ambiguity in the identification of the topological charges, we supplemented and verified the winding behavior of the topological charges at multiple different stages, while systematically improving the visualization of the polarization field throughout the process. Specifically, we provide (i) higher-density polarization maps and (ii) locally enlarged views around each topological charge for all stages in the Supplementary Note 3 and Fig.R2. These zoomed-in maps clearly reveal the continuous rotation of the polarization vector and confirm that the winding numbers of all charges are fully consistent with their assigned topological values in the main text.

Comment 3: The polarization ellipses around Figure S5d do not show clear winding behavior expected for the most upper and bottom charges.

Reply: We thank the Reviewer for this comment. While related to Comment 2, this point concerns a distinct physical situation-namely the fully merged state of topological charges.

In Fig. S5d, the topological charges in the central region have already undergone coaxial merging, forming a single radiation-suppressed node. In this regime, the individual charges are no longer spatially separated in momentum space, and their associated polarization vortices cannot be resolved independently in a discretely sampled polarization map. Instead, the polarization field reflects the combined topological charge, which in this case manifests as a single effective vortex. Therefore, the absence of clearly distinguishable winding patterns for the individual charges is not due to incorrect assignment, but is an intrinsic consequence of the merged configuration.

To make this point explicit, we have added an additional set of calculations in the Supplementary Note 6. In these calculations, we introduce a slight perturbation to the structural parameters, which gently lifts the exact merging condition and spatially separates the individual topological charges. Under this infinitesimal detuning, the polarization vortices associated with each charge become clearly visible, and their

winding behaviors are fully consistent with the assigned topological charges. This provides a direct verification of the charge composition prior to merging, and confirms that the merged state observed in Fig. S5d indeed originates from the coherent coalescence of multiple well-defined charges. The revised Fig. S5d is shown in Fig. R3.

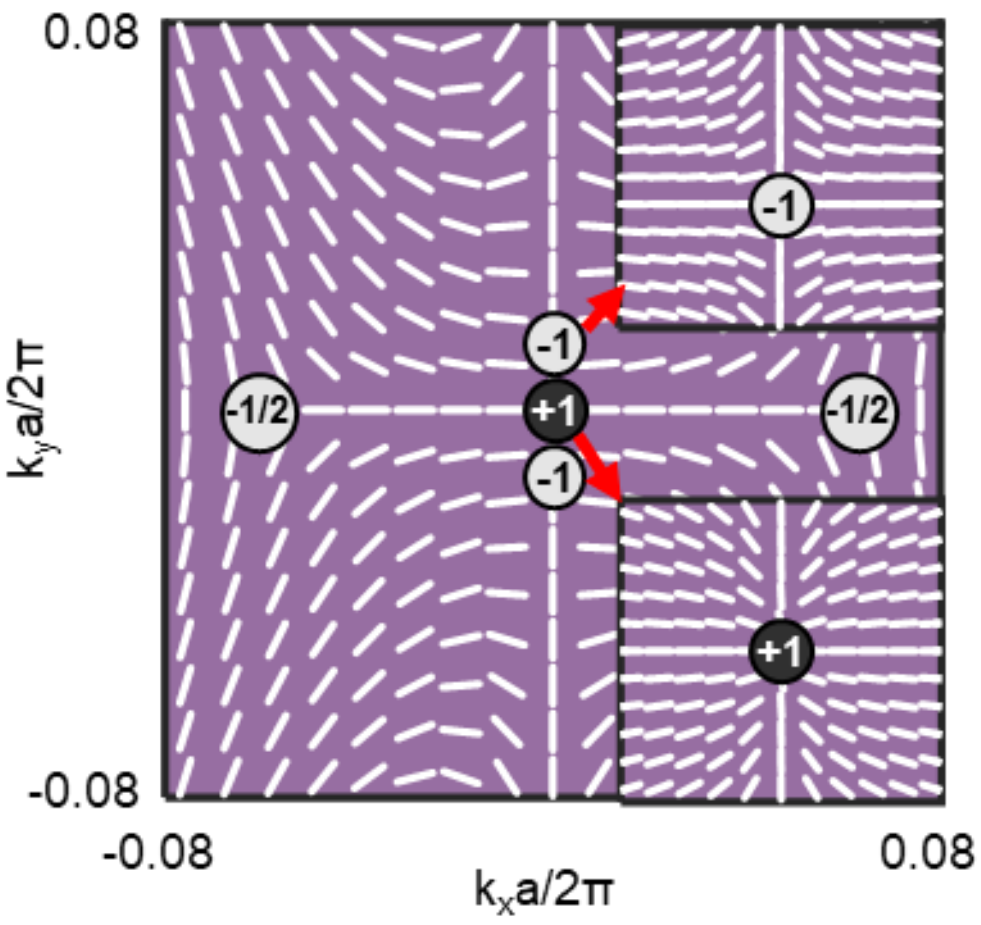

Figure R3 Schematics of the far-field polarization textures in momentum space for meging-BIC designs. The central panel shows an overall topological charge of −1, while the two insets display the polarization-vector configurations of the central and upper topological charges under slight parameter variations.

Comment 4: The line shapes for the spectra in Figure 4e, seem very far from a Lorentzian line shape and with much less noise than Figure S6.

Reply: We thank the Reviewer for this comment. We clarify that the apparent differences in line shape and noise level between Fig. 4e and Fig. S6 arise from the use of different measurement protocols, rather than from any change in the intrinsic resonance behavior of the device.

In our experiments, a tunable laser (Santec TSL-550, 1480–1630 nm) was used together with a photodiode (PDA10DT-EC) and a lock-in amplifier (SR830) for signal detection. For the measurements shown in Fig. S6, the sample was measured before substrate removal, where the scattered signal is relatively strong. In this case, the laser was operated in continuous sweep mode, and the lock-in amplifier integration time was

set to 1 ms to match the scan speed. The stronger signal intensity and shorter integration time introduced a higher signal fluctuations and noise level.

After substrate removal, however, the scattering signal becomes significantly weaker. To reliably resolve the resonance features (Fig. 4e), we switched to a step-scan mode with point-by-point acquisition and increased the lock-in integration time to 300 ms. This results in reduced apparent noise.

Importantly, we emphasize that these differences affect only the visual appearance of the spectra, not the extracted physical quantities. In both cases, the resonance positions and Q-factors are obtained through Lorentzian fitting, and we have verified that the extracted Q values are consistent within experimental uncertainty across different measurement configurations. We have added a description of these measurement conditions in the Methods section (lines 346-349) to clarify this point.

Response to Reviewer 2

Fu et al. demonstrated a BIC with a Q \prop k^-10 scaling law in a single-layer all-dielectric metasurface, and experimentally realized free-space Q-factors above 1x10^6 in the near-infrared regime. These performances are strikingly high compared with existing reports in the literature. They also demonstrated picometer-scale spectral discrimination making use of a 100-pixel metasurface array with such ultranarrow resonance. The results are very interesting and would be of great importance in the field of metasurfaces. However, before this manuscript can be considered for publication, the authors should carefully address the following problems.

Reply: We sincerely thank the Reviewer for the positive evaluation of our work and for the constructive and insightful comments. We are encouraged that the Reviewer recognizes the significance of the demonstrated ultrahigh-Q performance and picometer-scale spectral discrimination.

In response to the Reviewer's concerns, we have carefully revised the manuscript with a focus on three key aspects. First, we have clarified the topological charge evolution and merging mechanism, providing a complete and explicitly resolved momentum-space evolution to remove ambiguities in the original presentation. Second, we have refined the positioning of our work with respect to prior studies, including a more careful discussion of recent scaling results (e.g., k^{-8}) and a clearer delineation of the novelty and scope of our approach. Third, we have revised several experimental descriptions and claims, including robustness and measurement protocols, to ensure consistency, accuracy, and transparency.

We believe that these revisions have significantly strengthened the clarity, rigor, and presentation of the manuscript, and we hope that the revised version now meets the high standards of *Nature Communications*.

Comment 1: On the novelty. At first glance, the concept and the structure is very

similar to Kang et al. Light: Sci. Appl. 11, 228 (2022), which, unfortunately, was not cited by the authors. In that paper, photonic crystal slabs with a triangular lattice of cylindrical air holes were also adopted, and merging multiple BICs with a higher-order BIC (of higher-order topological charges) also greatly improves the Q factor with a k^-8 scaling law. The authors should comment that paper properly and clarify in details why Q~k^-10 can be obtained in this work but only k^-8 was obtained in that work. Accordingly, the novelty of this work should be rephrased since previously the authors only considered k^-6 as the upper limit in the literature.

Reply: We sincerely thank the Reviewer for pointing out the important work by Kang *et al.* (Light: Sci. Appl. 11, 228 (2022)). We fully agree that this study represents a significant advance in extending Q-scaling beyond conventional limits, and it should be properly discussed.

In our original submission, our discussion of prior work was primarily focused on experimentally demonstrated metasurface platforms, while the work by Kang *et al.* presents a theoretical framework for achieving higher-order scaling. This difference in focus contributed to an incomplete positioning of the state of the art, which we have now corrected. In the revised manuscript, we have incorporated this work into the Introduction (highlighted in red font in lines 69–74) and updated our description of the current scaling limits to explicitly include the $Q \propto k^{-8}$ result.

Importantly, we would like to clarify that the distinction between our work and previous studies is not simply the extension from k^{-8} to k^{-10}, but lies in the underlying physical mechanism governing the available topological charges. In previously reported cases (including both $Q \propto k^{-6}$ and $Q \propto k^{-8}$), the enhanced scaling is achieved through the merging of accidental BICs with a central symmetry-protected BIC, where the number of accessible charges remains constrained by the intrinsic band structure and symmetry. This constraint fundamentally limits the number of independent charge contributions that can participate in the merging process.

In contrast, our approach introduces a fundamentally different mechanism by

utilizing a high-order degenerate point as an independent topological charge reservoir. Through controlled degeneracy breaking, additional groups of integer and half-integer charges are released, reconfigured, and merged within a single band. This process effectively expands the available topological phase space beyond the conventional symmetry- and structure-limited framework.

As a result, our system enables a higher-order merged state with $Q \propto k^{-10}$ scaling, together with experimental realization of million-Q resonances and strong robustness against fabrication variations. We have added a detailed discussion in the revised manuscript (lines 187–191) to clarify why previous works were limited to $Q \propto k^{-8}$ and how our mechanism enables further scaling. Corresponding revisions have also been made in the Introduction (lines 84–89) and Conclusion (lines 302–307) to more accurately position the novelty of our work.

Comment 2: The authors should explicitly clarify how topological charge conservation is manifested during symmetry breaking and charge inversion.

Reply: We thank the Reviewer for this insightful comment. In the revised manuscript, we have now clarified how topological charge conservation is maintained during the symmetry-breaking and charge-evolution process both conceptually and visually.

Topological charge conservation in our system is governed by the continuity of the far-field polarization vector field in momentum space. During the evolution from the symmetric (C_6) to symmetry-broken configuration, no singularities are created or annihilated arbitrarily; instead, the total topological charge is strictly preserved through a combination of charge redistribution, inversion, and merging processes.

Specifically, two coupled processes occur simultaneously: (i) the outer accidental-BIC branch evolves continuously in momentum space, carrying its associated topological charges, and (ii) the lifting of the high-order degeneracy at Γ releases a set of hidden topological charges, including both integer and half-integer contributions.

The apparent "charge inversion" observed in the evolution is not a violation of conservation, but rather reflects a reconfiguration of the local polarization winding under symmetry breaking, where the orientation of the polarization vector field reverses while preserving the total winding number. Throughout this process, the global sum of all topological charges in the Brillouin zone remains invariant, consistent with topological conservation.

To make this process explicit, we have revised Figs. 2a–c by adding detailed annotations and have provided a complete, continuous evolution map of the polarization field in the Supplementary Note 3 and Fig.R1. This reconstruction clearly shows how all charges evolve, redistribute, and ultimately merge, while strictly satisfying charge conservation at every stage.

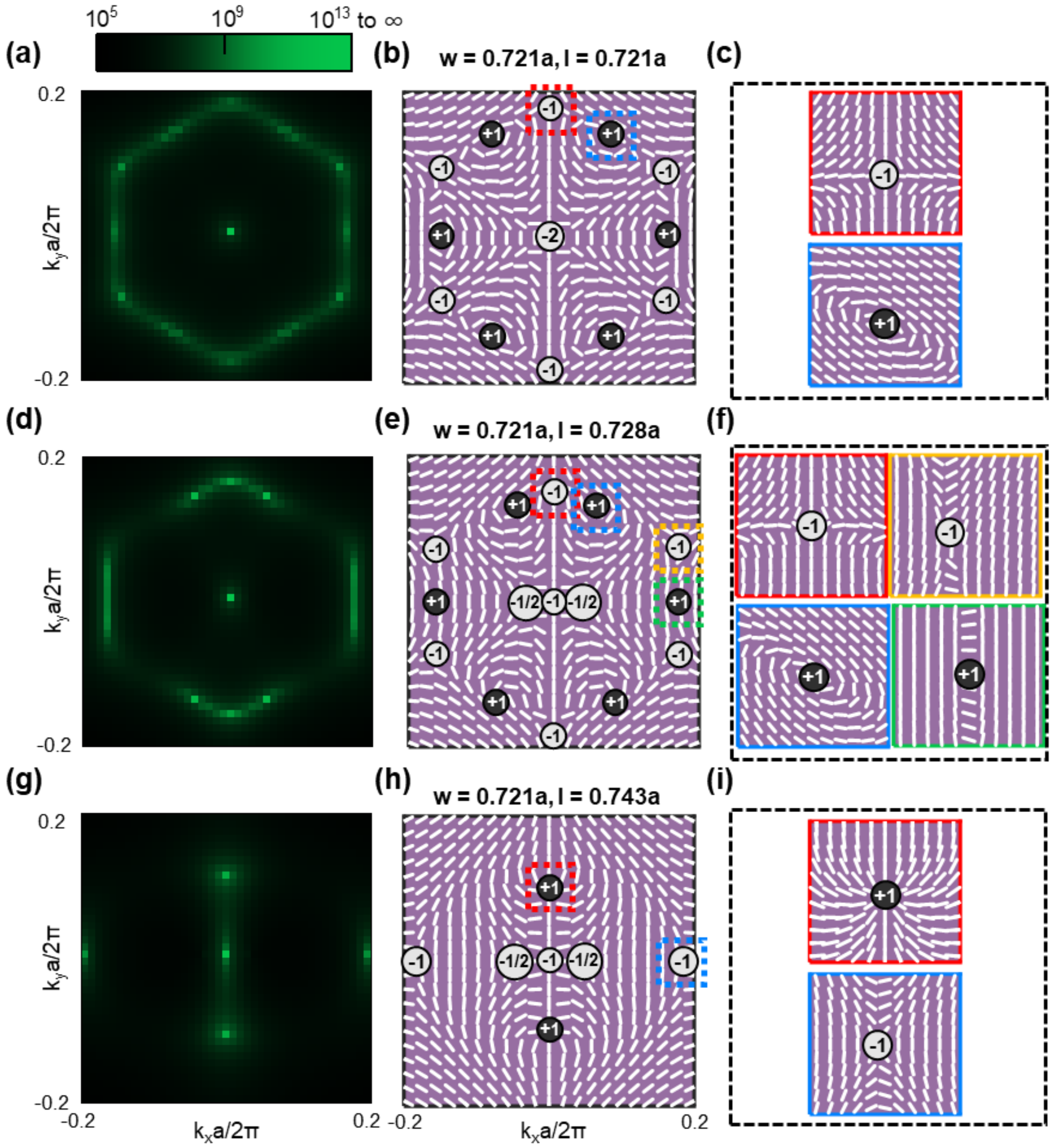

Figure R1 Simulated far-field polarization textures and Q-factor maps of the evolution of the outer-ring accidental-BIC topological charges during the symmetry-breaking process. Representative topological charges were selected according to the structural symmetry and enlarged to show their detailed polarization-vortex textures. **a, d, g**, Q-factor distribution map during the evolution process. **b, e, h**, Far-field polarization textures map during the evolution process. **c, f, i**, Locally enlarged views near the topological charges. The selected topological charges are marked with boxes of the same color.

Comment 3: In Fig. 4df, it is clear that Q factors for both k^-6 and k^-10 drop as ky*2*pi/a is smaller than 0.01 (approaching the Gamma point), but increase for k^-2. Why this happens?

Reply: We thank the Reviewer for this insightful question. The observed behavior arises from the interplay between the ideal scaling laws and practical experimental limitations, which affect different scaling regimes in distinct ways.

In an ideal system, all three cases follow the expected power-law scaling $Q \propto k^{-n}$, and the Q factor should increase monotonically as k approaches the Γ point. However, in realistic samples, two key factors modify this behavior:

First, the divergence of Q near Γ is truncated by fabrication imperfections and residual radiative loss, leading to a finite maximum Q rather than a true divergence. This effect is particularly pronounced for higher-order scaling (k^{-6} and k^{-10}), where the ideal Q increases very rapidly near Γ and is therefore more sensitive to disorder.

Second, in merging-BIC systems, the exact position of the merged topological charges may be slightly shifted away from Γ due to fabrication-induced asymmetry or small deviations from the ideal parameter set. As a result, the highest-Q region is not necessarily centered exactly at k=0, but instead forms a finite-width high-Q plateau around the nominal merging point.

Because of these two effects, the measured Q for k^{-6} and k^{-10} does not continue to increase arbitrarily as $k \to 0$, but instead shows a slight reduction or fluctuation near Γ, with the maximum Q occurring at a small but finite k. By contrast, in the k^{-2} case, the intrinsic scaling is much weaker, and the Q factor decreases rapidly with increasing k.

Therefore, within the experimentally accessible range, moving closer to Γ still results in a monotonic increase in Q, and the impact of the above two effects is less apparent.

We have clarified this point in the revised manuscript (highlighted in red font in lines 252-256) to better distinguish between the ideal scaling behavior and the experimentally observed Q distribution.

Comment 4: On the fabrication tolerance. In the Abstract, "free-space Q-factors above 1.1x10^6 together with +-3% geometric robustness" is overclaimed. According to this claim, the readers may expect Q factors above 1.1x10^6 for any geometric variations within +-3%. However, in Fig. 4d, only the short-axis width has 3% variation (not +-3%, which is 6% indeed), and all the samples (especially the red dots) have measured Q factors above 4x10^5, not 1.1x10^6.

Reply: We thank the Reviewer for this important comment and fully agree that the original wording in the Abstract was not sufficiently precise and could be misleading.

In the original statement, the phrase "free-space Q-factors above 1.1×10^6 together with ±3% geometric robustness" may indeed give the impression that Q-factors above 1.1×10^6 are maintained across the entire ±3% variation range, which is not what we intended to convey. Rather, our result demonstrates the coexistence of two distinct features: (i) a maximum Q-factor exceeding 1.1×10^6 under optimized conditions, and (ii) a high degree of robustness, where Q-factors remain consistently above ~10^5–10^6 across a finite range of geometric variations.

To avoid ambiguity, we have revised the statement in the Abstract (lines 39-41), Introduction (lines 90-91) and Conclusion (lines 307-309) to: "we realize exceptionally high free-space Q-factors together with 3% geometric robustness, with the maximum reaching 1.1×10^6" or similar phrasing.

We have also clarified in the main text that the robustness refers to the persistence of a high-Q plateau under geometric perturbations, rather than the preservation of the peak

Q value.

Comment 5: The authors fabricated samples based on a silicon-on-insulator wafer. They etched the oxide layer in order to ensure up-down mirror symmetry. However, the bottom silicon reflects strongly and degrade the transmission spectra, especially on the transmission efficiency when used for miniaturized spectrometers, although the Q factors are not changed. In this work, all the intensities in the measured spectra adopt arbitrary unit (a.u.), what are the original experimental data? Could the authors provide a few in the SM? Additionally, could the authors compare the simulated transmission spectra with and without the bottom silicon layer when the oxide layer is etched or not?

Reply: We thank the Reviewer for this insightful comment and for raising these important points regarding substrate effects and data presentation.

First, we clarify that the spectra reported in this work are obtained from reflection-side, resonance-enhanced scattering measurements, rather than transmission. Therefore, the device operation in our spectrometer demonstration does not rely on transmission efficiency through the structure, but instead on the spectral encoding of scattered intensity in free space. In this configuration, the presence of the bottom silicon primarily affects the background and collection efficiency, but does not alter the intrinsic resonance properties or the Q-factor.

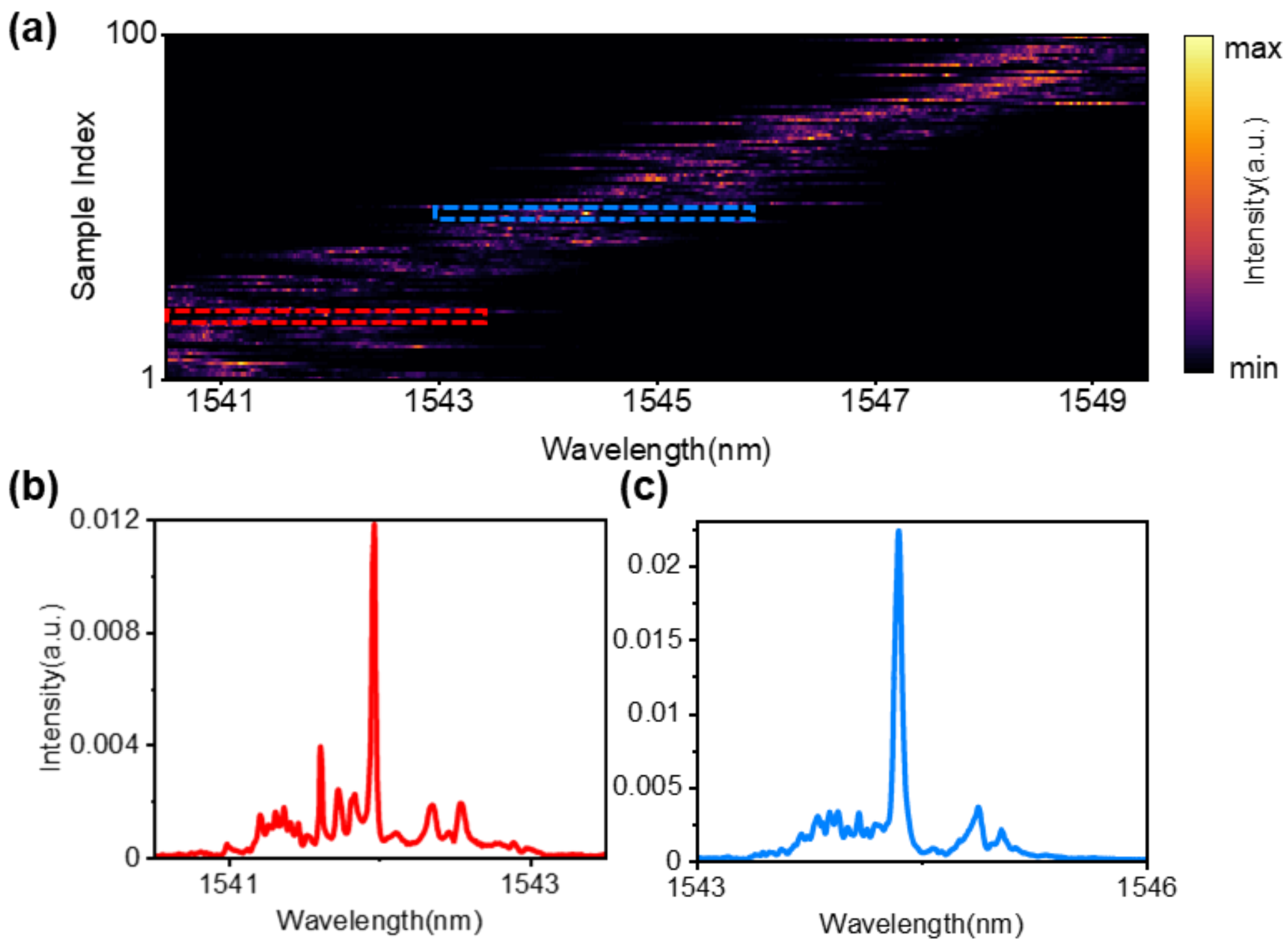

Figure R2 a, The response matrix corresponding to the spectral array composed of 100 samples. **b,** The specific scattering spectrum of the sample labeled in a.

Besides, as the Reviewer requests, in the Supplementary Note 8, we have made the following additions: (i) We provide representative raw experimental spectra as shown in Fig.R2 (prior to normalization) to clarify the origin of the a.u. intensity scale used in the main text. (ii) We have added a discussion clarifying that the spectrometer operation is based on free-space scattering rather than transmission filtering, and therefore is not limited by transmission loss associated with the substrate.

These additions aim to more clearly distinguish the measurement configuration and to address the Reviewer's concerns regarding both data interpretation and substrate effects.

Comment 6: In Fig. 2c, the half-integer topological charges should be more clearly indicated to improve clarity and reader comprehension.

Reply: We thank the reviewer for this helpful suggestion. In the revised manuscript, we have improved their visualization. Specifically, we have enlarged and explicitly labeled

all half-integer topological charges in Fig. 2c and all other relevant figures to enhance their visibility. In addition, we have refined the polarization-field representation and added clearer annotations to better highlight the local vortex structures associated with these charges. These modifications make the presence and role of half-integer charges in the merging process more explicit and easier to follow.

We sincerely thank the Reviewer again for the thoughtful and constructive comments, which have helped us significantly improve the clarity and rigor of the manuscript. We believe that all the concerns raised have been carefully addressed through substantial revisions in both the presentation and the positioning of our work. We hope that the revised manuscript now meets the reviewer's expectations and would be suitable for publication in *Nature Communications*.

Response to Reviewer 3

This manuscript investigates the topological reconfiguration mechanism of bound states in the continuum (BICs) induced by higher-order photonic degeneracies, and proposes as well as experimentally validates an implementation scheme for achieving ultrahigh-Q quasi-BICs based on an E2-type quadratic degeneracy. By introducing controlled symmetry breaking at the Γ point high-order degeneracy, the authors realize the release, inversion, and merging of multiple integer and half-integer topological charges. This approach surpasses the conventional scaling limit of $Q \propto k^{-n}$ ($n = 2$ to 6) typically observed in traditional BIC platforms, and establishes a new radiation-suppression scaling law of $Q \propto k^{-10}$. Experimentally, a free-space Q factor exceeding 1.1×10^6 is achieved. The quasi-BIC further maintains a high Q value under ±3% geometric perturbations, demonstrating strong robustness against fabrication imperfections. In addition, the authors showcase its application in high-resolution free-space spectral reconstruction, including the identification of $H^{13}C^{14}N$ absorption lines with picometer-level resolution. Overall, this work represents a significant technical breakthrough. In particular: (a) it achieves substantial progress in realizing ultrahigh-Q quasi-BICs; (b) the obtained quasi-BIC exhibits excellent tolerance to fabrication-induced perturbations; and (c) it demonstrates a free-space high-resolution spectroscopic system with promising application potential.

Despite these meaningful achievements in experimental implementation and performance, several theoretical aspects of the underlying mechanism still require further clarification and deeper analysis.

Reply: We sincerely thank the Reviewer for the highly positive evaluation of our work and for the insightful and constructive comments. We are particularly encouraged that the Reviewer recognizes the significance of our results in achieving ultrahigh-Q quasi-BICs, their robustness against fabrication perturbations, and their potential for high-resolution free-space spectroscopy.

We also appreciate the Reviewer's careful assessment that further clarification of the

underlying mechanism is needed. In response, we have carried out a series of detailed revisions aimed at strengthening the theoretical clarity and completeness of the manuscript. In particular, we have (i) clarified the physical origin and evolution of topological charges associated with the high-order degeneracy, (ii) provided a more explicit description of charge conservation, inversion, and merging processes in momentum space, and (iii) refined the scope and applicability of the $Q \propto k^{-n}$ scaling law within our framework. In addition, we have supplemented the manuscript with additional calculations and visualizations to make the topological evolution more transparent.

We believe that these revisions have substantially improved the rigor and clarity of the theoretical analysis, and that the revised manuscript now fully addresses the Reviewer's concerns.

Comment 1: On the Physics of "Higher-Order Degeneracy as a Topological Charge Reservoir"

1.1 Physical Origin of Accidental BICs under C_2 Symmetry is not clear. The authors propose that an E2-type quadratic degeneracy can release and reconstruct multiple integer and half-integer topological charges when the symmetry is reduced from C6 to C2. The manuscript provides a relatively detailed explanation on the topological property and BIC nature of the −2 charge at the Γ point, and we know the topological charge conservation under which the −2 charge splits into two −1 charges, one +1 charge, and two −1/2 charges during symmetry reduction, however, the physical origin of the two accidental BICs emerging along the k_x direction in the C2-symmetric structure remains insufficiently clarified.

Specifically, it is unclear whether these BICs arise from the reduced structural symmetry. If their origin is symmetry-based, why do they not exist in the C6-symmetric structure but emerge in the C2-symmetric configuration? Alternatively, if they are not directly dictated by symmetry but instead result from mode coupling or interband

interference mechanisms, the authors should provide a clear coupling model or an explicit physical picture to substantiate this interpretation.

Given that these accidental BICs play a crucial role in the subsequent multi-BIC merging process and in enabling the realization of ultrahigh-Q quasi-BICs, their physical origin warrants a more rigorous theoretical analysis rather than being supported solely by numerical observations.

Reply: We thank the Reviewer for this insightful and important question. We agree that the physical origin of these accidental BICs was not sufficiently clarified in the original manuscript, and we have now revised the text to provide a more explicit physical picture.

We would like to clarify that the two additional BICs observed along the k_y direction (It appears from the reviewer's description that you are referring to the two additional topological charges along the k_y direction; the mention of the k_x direction in the comment is likely a typographical error) in the C_2-symmetric structure are not newly generated by symmetry reduction itself, but instead originate from the outer-ring accidental-BIC branch that already exists in the band structure prior to merging, as shown in Fig. 2b. The role of symmetry breaking is therefore not to create these BICs, but to modify their evolution and interaction with the charges released from the lifted degeneracy at Γ.

Physically, these accidental BICs arise from destructive interference of radiation channels associated with the Bloch modes, and the polarization-singularity nature of BICs causes them to typically reside on high-symmetry lines of the structure. This is because the symmetry of a high-symmetry line enforces a definite polarization (s or p), thereby greatly reducing the degrees of freedom available to satisfy the destructive-interference condition among radiation channels. In the C_6-symmetric configuration, these states appear as a continuous ring of accidental BICs surrounding the Γ point along six high-symmetry lines according to symmetry. When the symmetry is reduced to C_2, this continuous ring is deformed and become concentrated on the new high-symmetry lines, and topological-charge conservation causes those not located on the

new symmetry lines to merge, leading to the emergence of distinct BICs along specific directions (e.g., the k_y axis).

Importantly, the symmetry reduction simultaneously lifts the high-order degeneracy at Γ, releasing additional topological charges. The key mechanism in our work is that these pre-existing accidental-BIC charges and the degeneracy-released charges coexist and subsequently interact, ultimately leading to the multi-charge merging process. Therefore, the accidental BICs are not solely symmetry-induced nor purely a byproduct of degeneracy lifting, but rather originate from radiation-channel cancellation within the band structure, whose evolution is reshaped by symmetry breaking.

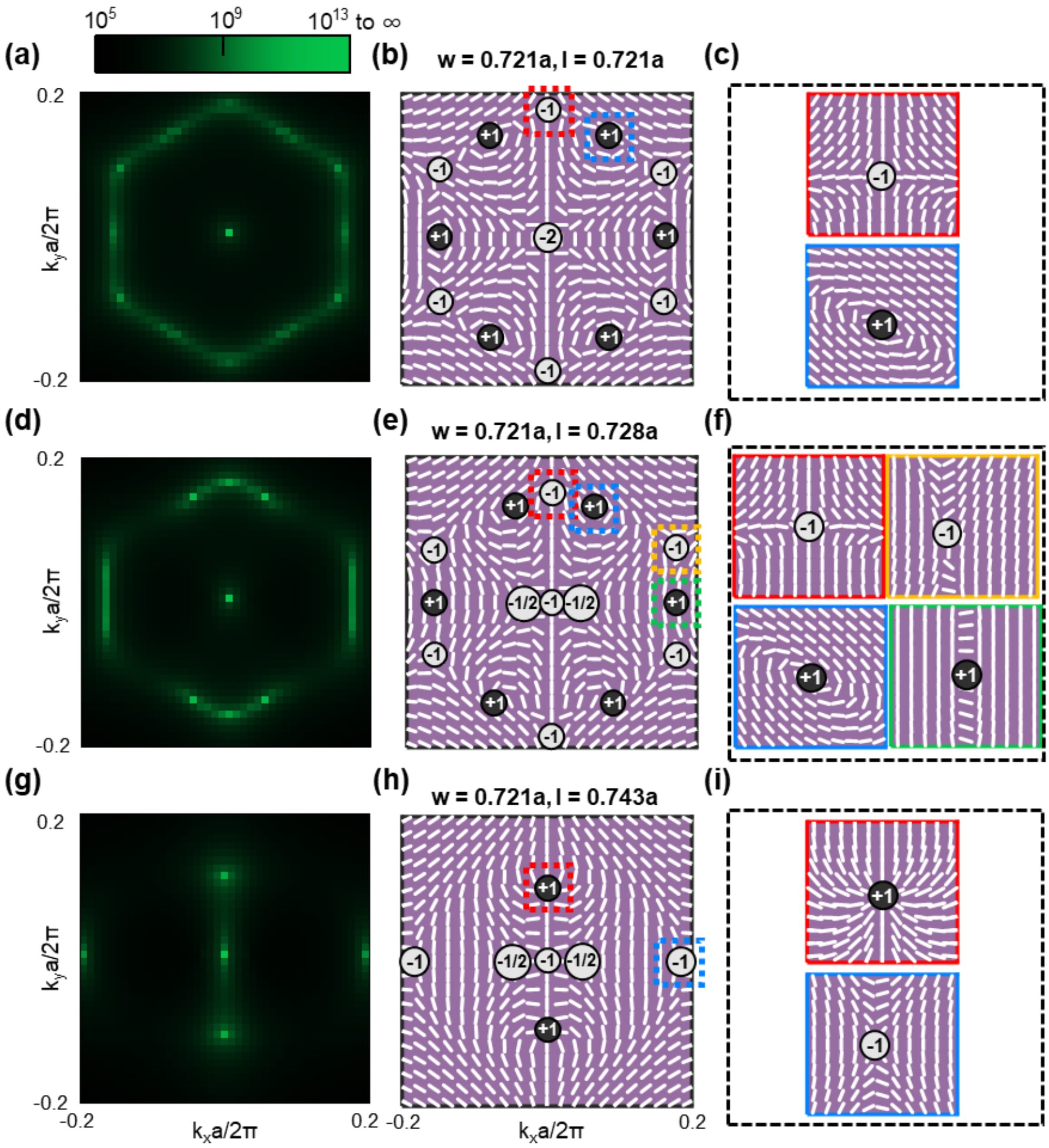

Figure R1 Simulated far-field polarization textures and Q-factor maps of the evolution of the outer-ring accidental-BIC topological charges during the symmetry-breaking process. Representative topological charges were selected according to the structural symmetry and enlarged to show their

detailed polarization-vortex textures. **a, d, g**, Q-factor distribution map during the evolution process. **b, e, h**, Far-field polarization textures map during the evolution process. **c, f, i**, Locally enlarged views near the topological charges. The selected topological charges are marked with boxes of the same color.

To make this mechanism clearer, we have revised the main text and added further detailed discussions (highlighted in red font in lines 161-178) to explicitly distinguish between the outer accidental-BIC branch and the degeneracy-induced charges, and we have provided a complete momentum-space evolution of these charges in the Supplementary Note 3 and Fig.R1. This presentation makes the origin, evolution, and eventual merging of all BICs unambiguous.

1.2 In light of the clarifications and refinements required for the issues discussed above, Figure 1(d) of the manuscript should be optimized and reconstructed to present, in a more rigorous and systematic manner, the global distribution and continuous evolution of topological charges throughout the entire symmetry-reduction process. Such improvement would enhance the completeness and logical coherence of the theoretical argumentation.

Reply: We thank the Reviewer for this valuable suggestion. We fully agree that a more systematic and continuous presentation of the topological-charge evolution would significantly improve the clarity and logical coherence of the manuscript.

In the revised manuscript, we have reconstructed Fig. 1(d) to provide a more complete and rigorous representation of the global topological-charge evolution throughout the symmetry-reduction process. In particular, the updated schematic now explicitly integrates: (i) the evolution of the outer-ring accidental-BIC charges, (ii) the release and redistribution of charges from the lifted high-order degeneracy at Γ, and (iii) the subsequent multi-charge merging process.

This revised figure (Fig.R2) presents a continuous and unified picture of the charge evolution in momentum space, clearly illustrating how different charge branches originate, evolve, and ultimately coalesce into the final high-order merged state. In this way, Fig.1d now serves as a consistent conceptual bridge between the physical

mechanism introduced in the Introduction and the detailed numerical analysis presented in Fig. 2.

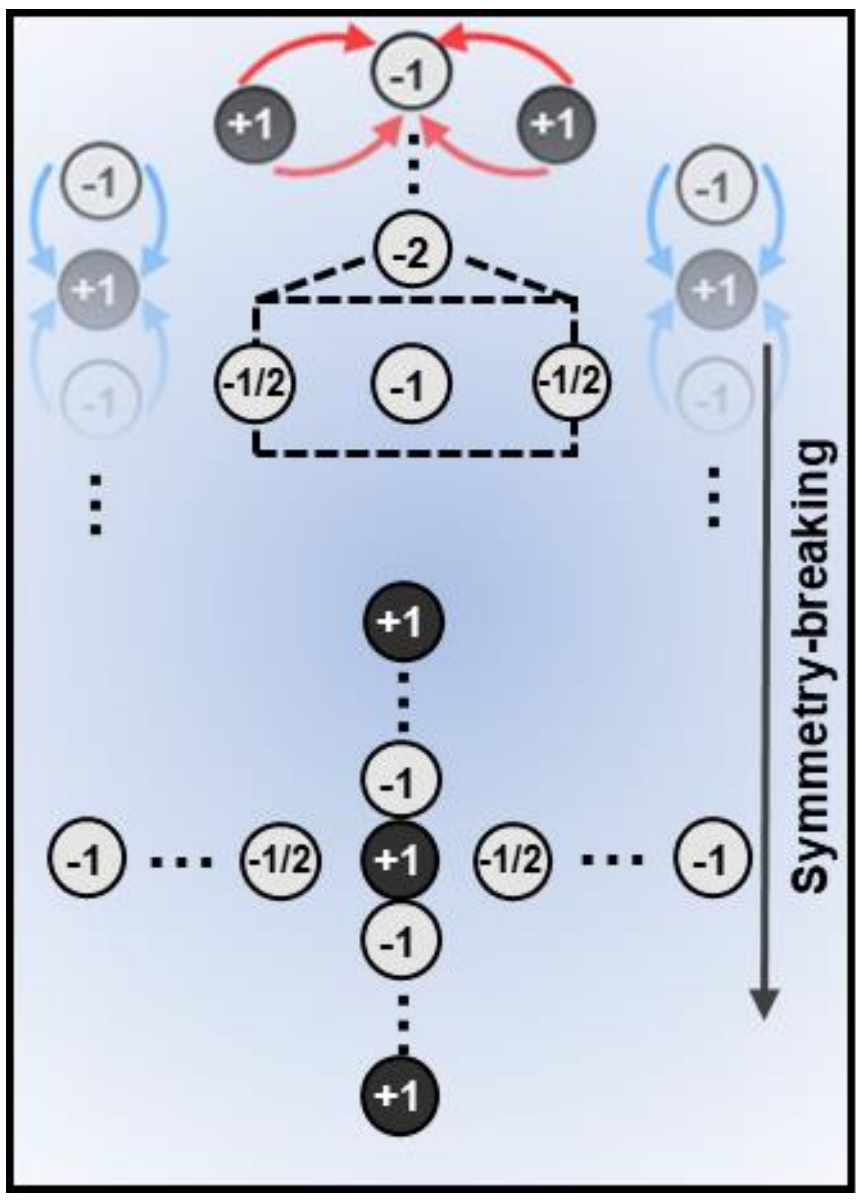

Figure R2 Schematic illustration of the evolution of topological charges during symmetry breaking. Symmetry breaking releases and redistributes multiple integer and half-integer topological charges, while also inducing the reorganization of the outer accidental BICs.

We believe that this reconstruction significantly improves the completeness and readability of the theoretical framework.

Comment 2: On the Derivation of the $Q \propto k^{-10}$ Scaling Induced by Multi-BIC Merging

2.1 Applicability and Generality of the Proposed Q－k Scaling Law. The authors claim to have derived a Q－k scaling law applicable to “degenerate-point BICs.” It remains necessary to clarify whether this derivation can also be extended to previously reported merging BIC scenarios (i.e., BIC merging occurring on non-degenerate bands). In other words, is the proposed merging mechanism universal, or is it restricted to the specific TM3 mode and higher-order degeneracy configuration discussed in this work? The authors are encouraged to more clearly delineate the scope and applicability of their theoretical framework.

Furthermore, the authors state that “when k_"BIC1" and k_"BIC2" both approach

zero, the approximation leads to Q ∝ 1/ky^(10)." However, neither the main text nor the Supplementary Material clearly presents the full merging process in momentum space, i.e., the continuous evolution of BICs toward the Γ point along the ky direction. This momentum-space merging trajectory is central to substantiating the core claim that multi-charge merging results in tenth-order scaling. A systematic presentation is therefore required, including: 1. Continuous evolution maps of BIC positions as structural parameters vary; 2. The corresponding evolution of the scaling exponent during the merging process.

Reply: We thank the Reviewer for this important and insightful comment. We have revised the manuscript to more clearly delineate both the scope of applicability of our Q-k scaling law and the momentum-space evolution process underlying the multi-BIC merging mechanism.

First, regarding the generality of the proposed scaling law, we clarify that the derivation of $Q \propto k^{-10}$ in this work is built upon the standard theoretical framework of merged BICs, where the radiative loss is governed by the collective interference of multiple topological charges. In the final stage of our system, the symmetry is already broken and the merging occurs in a non-degenerate regime, making the derivation formally consistent with previously reported merging-BIC scenarios on non-degenerate bands.

The key distinction of our work lies not in a fundamentally different derivation, but in the presence of an additional, independently generated group of topological charges, originating from the lifted high-order degeneracy. This effectively extends the conventional merging-BIC framework by introducing an extra topological degree of freedom, which enables higher-order scaling ($Q \propto k^{-10}$). Therefore, the underlying theory is not universal to all BIC systems, but is generally applicable to systems in which multiple charge families—particularly those arising from degeneracy-induced charge reservoirs—can be simultaneously controlled and merged. We have added this explanation to the corresponding derivation in Supplementary Note 4.

Second, regarding the momentum-space merging trajectory, we agree that a clear and continuous description is essential to substantiate the scaling behavior. In the revised manuscript, we have provided a systematic presentation of the full merging process in the Supplementary Note 3, including: (i) continuous evolution maps of BIC positions in momentum space as structural parameters vary (Fig.R1), and (ii) the corresponding evolution of the Q–k scaling behavior throughout the merging process. (Fig.R3)

These results explicitly show how the initially separated BICs approach each other and progressively merge toward the Γ point, and how the scaling exponent evolves from lower-order behavior (e.g., k^{-2}) to the final $Q \propto k^{-10}$ regime. This complete evolution provides direct support for the physical origin of the tenth-order scaling. This further clarifies that the k^{-10} scaling is not an isolated result, but the natural outcome of a continuous merging process involving multiple charge families.

We have revised the manuscript accordingly to clearly define the applicability of the theory and to present the merging trajectory in a more rigorous and transparent manner.

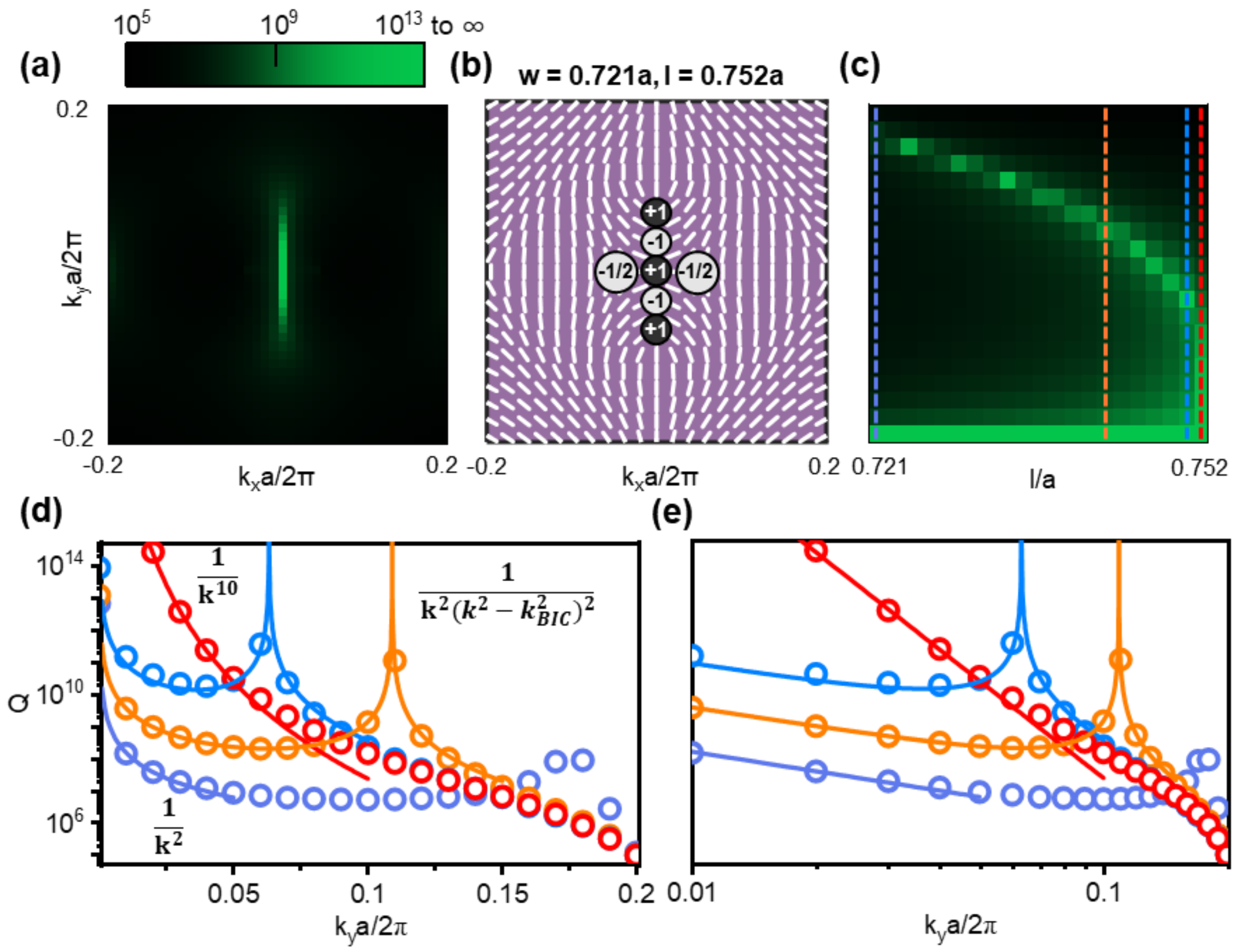

Figure R3 a, b Q-factor distribution map and far-field polarization textures map of the final merged

state. **c,** Variation of the Q-factor distribution along the k_y axis as symmetry is broken. **d, e,** As the structural symmetry is broken, the scaling law of the Q factor changes. The curves in different colors correspond to the different states labeled in c. As the system evolves, the scaling behavior changes step by step from k^{-2} to $Q \propto k^{-10}$. Under the current parameter settings, when the accidental BIC in the outer region merges with the central one, the central degeneracy point simultaneously releases the hidden BIC inside, resulting in a $Q \propto k^{-10}$ scaling behavior.

2.2 On the Experimental Q Factor at the Order of Million. The experimentally reported Q factor on the order of 106 raises an additional question: is this value already approaching the measurement limit of the experimental system, or does it genuinely reflect the intrinsic radiation suppression enabled by the proposed higher-order degeneracy and multi-charge merging mechanism?

Reply: We thank the Reviewer for this important question. We have carefully evaluated whether the experimentally observed Q-factor is limited by the measurement system or reflects the intrinsic performance of the device.

In our experiments, a tunable laser (Santec TSL-550, 1480–1630 nm) is used, which provides a wavelength tuning precision of 0.0001 nm and a linewidth of approximately 200 kHz (≈0.00016 nm). This spectral resolution is significantly narrower than the measured resonance linewidths corresponding to $Q \sim 10^6$, indicating that the measurement system is fully capable of resolving even higher-Q resonances.

Therefore, we conclude that the experimentally obtained Q-factors are not limited by the instrumental resolution, but are instead primarily determined by the intrinsic radiative suppression mechanism together with residual fabrication imperfections and material losses. In particular, deviations from the ideal merging condition and unavoidable structural disorder truncate the ideal divergence of Q near the Γ point, leading to the observed finite but ultrahigh-Q values.

We have clarified this point in the Methods section (lines 337-338) to better distinguish between measurement limitations and the intrinsic device performance.

In summary, this work represents a significant technical breakthrough in the experimental realization and application of ultrahigh-Q quasi-BICs. In particular, achieving Q factors at the 10^6 level on a free-space, single-layer dielectric platform with

high fabrication tolerance is of substantial importance. We recommend that the manuscript be considered for publication after the authors provide thorough clarification and strengthened theoretical justification regarding the core physical issues outlined above.

Reply: We sincerely thank the Reviewer for the highly positive evaluation of our work and for the thoughtful and constructive comments. We are particularly encouraged by the recognition of the significance of achieving ultrahigh-Q quasi-BICs with strong robustness in a free-space, single-layer platform.

In response to the Reviewer's comments, we have carefully revised the manuscript to address all the key concerns raised, with particular emphasis on strengthening the theoretical clarity and rigor. Specifically, we have clarified the physical origin of the accidental BICs, provided a complete and continuous description of the topological charge evolution and merging process, and refined the scope and applicability of the proposed Q–k scaling law. These revisions are supported by additional calculations and improved visualizations to ensure a consistent and transparent physical picture.

We believe that these revisions could address the Reviewer's concerns and significantly improved the clarity and completeness of the manuscript. We hope that the revised version is now suitable for publication in *Nature Communications*.

This manuscript investigates the topological reconfiguration mechanism of bound states in the continuum (BICs) induced by higher-order photonic degeneracies, and proposes as well as experimentally validates an implementation scheme for achieving ultrahigh-Q quasi-BICs based on an E_2-type quadratic degeneracy. By introducing controlled symmetry breaking at the Γ point high-order degeneracy, the authors realize the release, inversion, and merging of multiple integer and half-integer topological charges. This approach surpasses the conventional scaling limit of $Q \propto k^{-n}$ (n = 2 to 6) typically observed in traditional BIC platforms, and establishes a new radiation-suppression scaling law of $Q \propto k^{-10}$. Experimentally, a free-space Q factor exceeding 1.1×10^6 is achieved. The quasi-BIC further maintains a high Q value under ±3% geometric perturbations, demonstrating strong robustness against fabrication imperfections. In addition, the authors showcase its application in high-resolution free-space spectral reconstruction, including the identification of $H^{13}C^{14}N$ absorption lines with picometer-level resolution. Overall, this work represents a significant technical breakthrough. In particular: (a) it achieves substantial progress in realizing ultrahigh-Q quasi-BICs; (b) the obtained quasi-BIC exhibits excellent tolerance to fabrication-induced perturbations; and (c) it demonstrates a free-space high-resolution spectroscopic system with promising application potential.

Despite these meaningful achievements in experimental implementation and performance, several theoretical aspects of the underlying mechanism still require further clarification and deeper analysis.

I. On the Physicse of "Higher-Order Degeneracy as a Topological Charge Reservoir"

1.1 Physical Origin of Accidental BICs under C_2 Symmetry is not clear. The authors propose that an E_2-type quadratic degeneracy can release and reconstruct multiple integer and half-integer topological charges when the symmetry is reduced from C_6 to C_2. The manuscript provides a relatively detailed explanation on the topological property and BIC nature of the −2 charge at the Γ point, and we know the topological charge conservation under which the −2 charge splits into two −1 charges, one +1 charge, and two −1/2 charges during symmetry reduction, however, the physical origin of the two accidental BICs emerging along the k_x direction in the C_2-symmetric structure remains insufficiently clarified.

Specifically, it is unclear whether these BICs arise from the reduced structural symmetry. If their origin is symmetry-based, why do they not exist in the C_6-symmetric

structure but emerge in the C_2-symmetric configuration? Alternatively, if they are not directly dictated by symmetry but instead result from mode coupling or interband interference mechanisms, the authors should provide a clear coupling model or an explicit physical picture to substantiate this interpretation.

Given that these accidental BICs play a crucial role in the subsequent multi-BIC merging process and in enabling the realization of ultrahigh-Q quasi-BICs, their physical origin warrants a more rigorous theoretical analysis rather than being supported solely by numerical observations.

1.2 In light of the clarifications and refinements required for the issues discussed above, Figure 1(d) of the manuscript should be optimized and reconstructed to present, in a more rigorous and systematic manner, the global distribution and continuous evolution of topological charges throughout the entire symmetry-reduction process. Such improvement would enhance the completeness and logical coherence of the theoretical argumentation.

II. On the Derivation of the $Q \propto k^{-10}$ Scaling Induced by Multi-BIC Merging

2.1 Applicability and Generality of the Proposed Q–k Scaling Law. The authors claim to have derived a *Q–k* scaling law applicable to “degenerate-point BICs.” It remains necessary to clarify whether this derivation can also be extended to previously reported merging BIC scenarios (i.e., BIC merging occurring on non-degenerate bands). In other words, is the proposed merging mechanism universal, or is it restricted to the specific TM_3 mode and higher-order degeneracy configuration discussed in this work? The authors are encouraged to more clearly delineate the scope and applicability of their theoretical framework.

Furthermore, the authors state that “when k_{BIC1} and k_{BIC2} both approach zero, the approximation leads to $Q \propto 1/k_y$^(10).” However, neither the main text nor the Supplementary Material clearly presents the full merging process in momentum space, i.e., the continuous evolution of BICs toward the Γ point along the k_y direction. This momentum-space merging trajectory is central to substantiating the core claim that multi-charge merging results in tenth-order scaling. A systematic presentation is therefore required, including: 1. Continuous evolution maps of BIC positions as structural parameters vary; 2. The corresponding evolution of the scaling exponent during the merging process.

2.2 On the Experimental Q Factor at the Order of Million. The experimentally reported Q factor on the order of 10^6 raises an additional question: is this value already

approaching the measurement limit of the experimental system, or does it genuinely reflect the intrinsic radiation suppression enabled by the proposed higher-order degeneracy and multi-charge merging mechanism?

In summary, this work represents a significant technical breakthrough in the experimental realization and application of ultrahigh-Q quasi-BICs. In particular, achieving Q factors at the 10^6 level on a free-space, single-layer dielectric platform with high fabrication tolerance is of substantial importance. We recommend that the manuscript be considered for publication after the authors provide thorough clarification and strengthened theoretical justification regarding the core physical issues outlined above.
